# Linking the effects of helminth infection, diet and the gut microbiota with human whole-blood signatures

**DOI:** 10.1371/journal.ppat.1008066

**Published:** 2019-12-16

**Authors:** Soo Ching Lee, Mei San Tang, Alice V. Easton, Joseph Cooper Devlin, Ling Ling Chua, Ilseung Cho, Foong Ming Moy, Tsung Fei Khang, Yvonne A. L. Lim, P’ng Loke

**Affiliations:** 1 Department of Parasitology, Faculty of Medicine, University of Malaya, Kuala Lumpur, Malaysia; 2 Centre of Excellence for Research in AIDS (CERiA), University of Malaya, Kuala Lumpur, Malaysia; 3 Department of Microbiology, New York University School of Medicine, New York, New York, United States of America; 4 Department of Obstetrics and Gynecology, Faculty of Medicine, University of Malaya Medical Centre, Kuala Lumpur, Malaysia; 5 Department of Paediatrics, Faculty of Medicine, University of Malaya Medical Centre, Kuala Lumpur, Malaysia; 6 Department of Medicine, Division of Gastroenterology, New York University School of Medicine, New York, New York, United States of America; 7 Department of Social and Preventive Medicine, Faculty of Medicine, University of Malaya, Kuala Lumpur, Malaysia; 8 University of Malaya Centre for Data Analytics, University of Malaya, Kuala Lumpur, Malaysia; 9 Institute of Mathematical Sciences, Faculty of Science, University of Malaya, Kuala Lumpur, Malaysia; Washington University School of Medicine, UNITED STATES

## Abstract

Helminth infection and dietary intake can affect the intestinal microbiota, as well as the immune system. Here we analyzed the relationship between fecal microbiota and blood profiles of indigenous Malaysians, referred to locally as Orang Asli, in comparison to urban participants from the capital city of Malaysia, Kuala Lumpur. We found that helminth infections had a larger effect on gut microbial composition than did dietary intake or blood profiles. *Trichuris trichiura* infection intensity also had the strongest association with blood transcriptional profiles. By characterizing paired longitudinal samples collected before and after deworming treatment, we determined that changes in serum zinc and iron levels among the Orang Asli were driven by changes in helminth infection status, independent of dietary metal intake. Serum zinc and iron levels were associated with changes in the abundance of several microbial taxa. Hence, there is considerable interplay between helminths, micronutrients and the microbiota on the regulation of immune responses in humans.

## Author summary

Parasitic intestinal worms and gut bacteria occupy the same space, but we do not understand the nature and scope of their interaction. This is further complicated by dietary effects on the gut bacteria, as well as the immune responses of the host. To better understand these complex interactions, we compared individuals living in indigenous communities in Malaysia, where worm infections are common, with people living in the capital of Malaysia, who were not infected with worms. Data collected included burden of infection, a dietary survey, clinical tests, RNA profiles on blood samples and gut bacteria composition. By collecting data before and after treating the indigenous Malaysians with deworming medication, we could determine what was associated with changes in worm burden following deworming. We found that worm infection had a larger effect on gut bacteria composition than did dietary intake or blood profiles. Worm burden also had the strongest association with blood RNA profiles. We found that zinc and iron levels in the blood were associated with changes in helminth infection status, independent of dietary metal intake. Our results suggest that there is considerable interplay between intestinal worms and gut bacteria with zinc and iron levels in infected people.

## Introduction

During the course of human evolution, the majority of our ancestors were likely colonized by helminths, as are most mammals in the wild [1]. We have thus co-evolved to tolerate their colonization and to minimize their virulence [2]. Genes have been selected on both sides to optimize what is almost a biotic partnership [3]. However, the heterogeneity in responses to helminths has led to severe morbidity in a proportion of infected individuals [3]. This heterogeneity is poorly understood and could be the result of a complex relationship between host genotype, parasite genotype and also the gut microbiota of infected individuals.

The most widespread helminth infections in man, as well as in most other mammals, are the intestinal helminths [4–6]. This is also the locale of the majority of commensal bacteria in mammals. Undoubtedly, there must be important interactions among these organisms residing in the same environmental niche [7, 8], but these are poorly understood. Recent data that others [9–11] and we [8, 12, 13] have generated indicate that helminths can affect microbial diversity and composition of the gut microbiota. Notably, successful colonization by *Trichuris muris* in the large intestine of mice is dependent on the microbiota [14]. Hence, helminths can influence the microbiota communities and the microbiota can influence helminth infectivity. The microbiota can also alter the efficacy and toxicity of drugs [15], hence may impact the pharmacodynamics of deworming treatment.

Whilst previous generations faced predominant threats of infectious agents [16], developed countries now face an epidemic of diseases associated with dysregulated inflammation [17]. The rapid time frame for the ascent of these diseases points towards alterations in the environment as a cause. Differences in the microbiota of populations in developing and developed countries [18], and the eradication of helminth infections [19], may be environmental factors contributing towards these epidemiological trends that are relevant to the hygiene hypothesis [20] or “Old Friends” hypothesis [21]. In order to avoid rejection, helminths have evolved mechanisms to regulate the immune system of their hosts [22], including production of various immune-regulatory molecules [23] encoded into their genomes [24]. Concurrently, it is now recognized that there are many immunological effects of the microbiota on their mammalian hosts [25], especially important in regulating the intestinal immune system [26–28]. Shifts in these bacterial communities may alter the immune response of their hosts and lead to increased inflammatory responses [29]. Given that composition of bacterial communities is altered by helminth infections [9, 11, 13, 30], the absence of helminth infections in developed countries may be responsible for a subset of these shifts.

We have found that helminth infections can reverse dysbiosis and improve symptoms in animal models of inflammatory bowel diseases [12, 31] (IBD). Mice deficient in the IBD susceptibility gene *Nod2* are protected from intestinal abnormalities [32] by helminth infections through inhibiting colonization by an inflammatory *Bacteroides* species [31]. Resistance to *Bacteroides* colonization was dependent on type 2 immunity, which promotes the establishment of a protective microbiota enriched in Clostridiales. Additionally, we showed that indigenous individuals from Malaysia called the Orang Asli harbor a similar protective microbiota and that deworming treatment reduced levels of Clostridiales and increased Bacteroidales [31]. Certain individuals may therefore be more genetically susceptible to the consequences of a changing microbial environment.

Since the relative contribution of diet and helminth infections on the inter-individual variation of microbial communities has not been assessed, we conducted nutritional surveys on helminth infected individuals living in a rural setting, as well as urban participants. We also utilized whole-blood transcriptional profiling, and assessed a large panel of clinical variables from the blood, to characterize the relationships between peripheral blood variables with gut microbial communities and helminth infection. We assessed changes to blood transcriptional profiles and serum markers after deworming treatment of the helminth-infected individuals. The longitudinal analysis of these paired samples enabled us to assess which of the correlated variables were likely to be a consequence of helminth infections. We identified serum zinc levels as being associated with helminth infections; increased zinc levels may be induced directly by helminths or by microbial communities promoted by helminth infection. Thus, we gained new insights into the complex interplay between helminth infections and microbial factors that affect human micronutrient regulation and immune responses.

## Results

### Overview of the study

**Fig 1:**
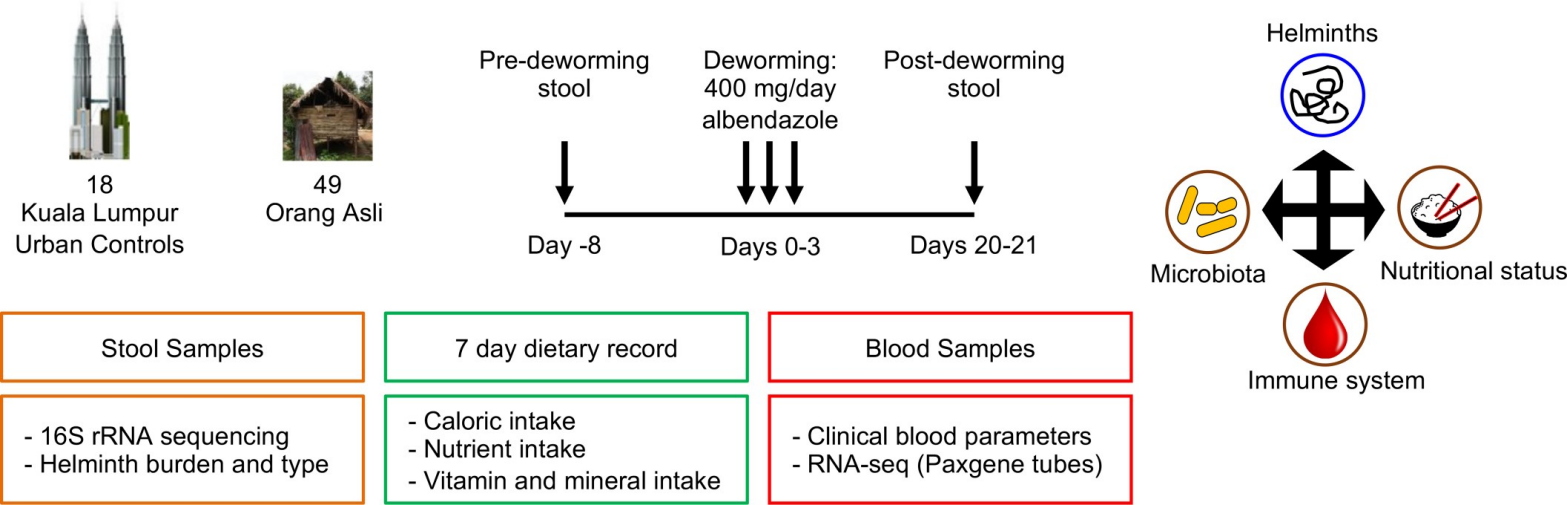
Study design. This study consists of a cross-sectional and a longitudinal phase. The cross-sectional phase compared between data sets generated from urban participants and Orang Asli participants. Eighteen residents of Kuala Lumpur, who served as urban control participants, provided stool specimens for microbial 16S rRNA profiling, blood specimens (for measurements of clinically relevant indices and whole-blood transcriptional profiling) and nutritional survey data. Forty-nine Orang Asli responded to a nutritional questionnaire and provided a blood specimen (for measurements of clinically relevant indices and/or whole-blood transcriptional profiling, depending on the volume of blood specimen collected). Forty of these Orang Asli participants also provided a stool specimen. During the longitudinal phase, Orang Asli participants were treated with 400mg albendazole daily over three days, with blood and/or stool specimen collection again 20–21 days post-deworming. We were able to follow up with 42 Orang Asli participants and obtained paired blood specimens (pre- and post-deworming) for whole blood transcriptional profiling. Approximately 70% of these 42 dewormed Orang Asli participants provided additional blood and/or stool specimen(s) for longitudinal analysis of clinically relevant blood indices and/or 16S rRNA profiles. Variation in the number of samples for each analysis are detailed in **S2 Table**.

This study consists of a cross-sectional component that examined the biological differences between urban residents (individuals with lifestyles that resembled that of residents of more developed countries) and indigenous individuals (individuals generally regarded to be resistant to autoimmunity), as well as a longitudinal component that examined the biological changes induced by deworming (**Fig 1**). The cross-sectional component sought to identify potential physiological variables that would confer resistance to autoimmunity among the indigenous individuals, while the longitudinal component aimed to identify biological consequences that occurred over a period associated with the loss of intestinal helminth colonization.

During the cross-sectional phase of the study, we first conducted a detailed 7-day nutritional questionnaire among 49 consenting asymptomatic Malaysian indigenous (hereafter referred to as “Orang Asli”) individuals living in a rural setting next to the jungle to determine dietary intake variables. We also collected blood samples from these consenting individuals into PAXgene (Qiagen, Valencia, CA) tubes for genome-wide whole-blood transcriptional profiling by RNA-seq analysis, as well as for measurements of additional hematological variables and serum chemistries when the blood specimen was of adequate volume. The additional clinical variables measured included (1) complete blood counts, (2) serum chemistries that examine an individual’s renal function, liver function and metabolic state, as well as (3) serum levels of metals with clinical relevance (**S1 Table**). Stool samples were also collected for quantification of helminth infection type and burden, as well as to generate microbial profiles by 16S rRNA gene sequencing. There were 40 participants for whom we generated a complete set of nutritional survey, whole blood transcriptional profiling by RNA-seq, blood clinical measurements and stool 16S rRNA profiling. Incomplete records were collected for some participants due to insufficient volumes of blood and stool samples being collected, or loss to follow-up, leading to minor variations in the number of samples included in each of the analyses performed in this study (detailed in **S2 Table**). For comparison, we collected similar sets of nutritional, stool microbiota and blood clinical/transcriptional data from 18 individuals from Malaysia’s capital city (Kuala Lumpur) at a single timepoint, who served as baseline urban control participants.

The longitudinal phase of our study began approximately 1 week after the collection of the samples for cross-sectional analyses. The same cohort of 49 Orang Asli participants, who provided consent during the cross-sectional phase described above, were treated with deworming medication (3 doses of albendazole 400mg/day). Approximately 3 weeks later, blood and stool samples were again collected for profiling of variables similar to those described in the cross-sectional study above. We were able to follow up with 42 of the individuals who received deworming treatment and obtained paired post-deworming PAXgene blood samples from these individuals. In addition, approximately seventy percent of these post-dewormed individuals provided additional blood and/or stool samples for post-deworming 16S rRNA profiling and/or measurement of serum chemistries. Again, there were minor variations in the number of subjects analyzed for different comparisons (detailed in **S2 Table**), depending on the different combinations of blood and/or stool samples that were obtained from each individual. Nonetheless, we could still identify biological variables that responded to deworming in both the stool and blood samples using the paired pre- and post-deworming samples from these individuals. Urban participants were not treated and thus only had samples collected at one time point during the cross-sectional phase of the study. Demographic and infection-status characteristics of study participants are summarized in **S3 Table**.

### Differences between the Orang Asli and urban participants

**Fig 2:**
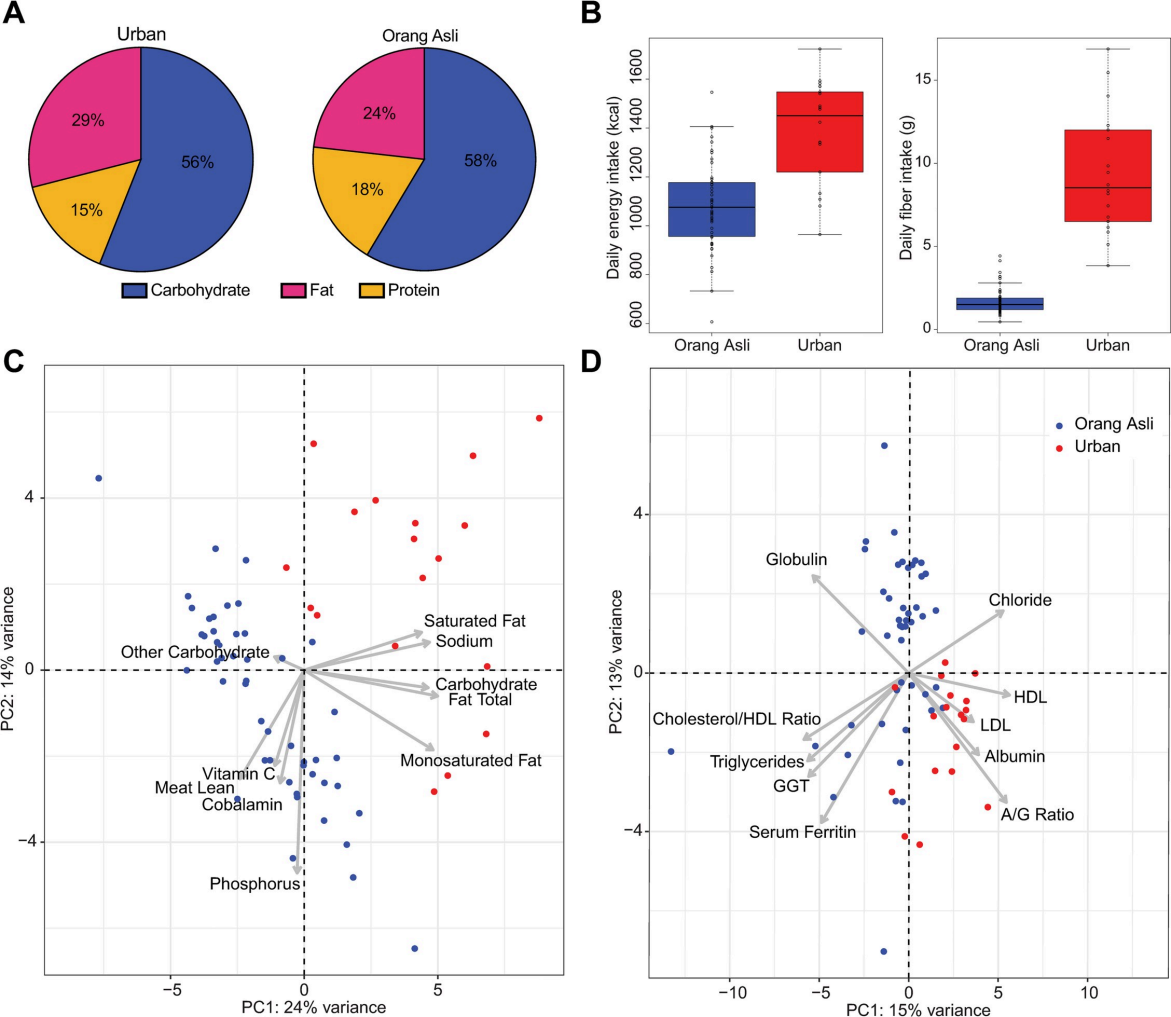
Dietary intake and nutritional status of study participants. (A) The proportion of dietary calories from carbohydrate, protein and fat among 49 Orang Asli (right) and 18 urban participants (left). (B) Daily energy intake in kilocalories was significantly higher (two-sample t-test, p<0.0001) in the urban study population (1386 ± 50) than in Orang Asli participants (1082 ± 26). Similarly, daily fiber intake (in grams) was significantly higher (two-sample t-test, p<0.0001) in the urban study population (9.35 ± 0.86) than in Orang Asli participants (1.74 ± 0.12). PCA based on (C) nutritional profiles from these 49 Orang Asli and 18 urban participants. (D) PCA based on blood variables from 46 Orang Asli and 18 urban participants. PCA is visualized as biplots, where data points represent PCA scores of individual samples while arrows represent loading values, which indicate the direction and magnitude of association between individual variables and PCs 1–2. Only nutritional and blood variables with the five most positive and most negative loading values on PC 1 are visualized.

We first analyzed inter-individual differences for dietary intake, clinical blood variables and stool microbial profiles between the Orang Asli and the urban control participants (**Fig 2**). A 7-day dietary record provided a view into the differences between the dietary habits of the Orang Asli and urban participants, showing the fraction contributed by each food source to their overall energy intake (**Fig 2A**), as well as micro and macronutrient intakes. This enabled a caloric breakdown, which determined that the Orang Asli obtained a larger fraction of their overall energy intake through protein (18% of total caloric intake) as compared to the urban participants (15% of total caloric intake: p < 0.0001) (**Fig 2A**). Overall, the urban controls had a significantly higher caloric intake than the Orang Asli (1386 ± 50 versus 1082 ± 26, two-sample t-test, p = 9.60 × 10^−7^) and fiber consumption (9.35 ± 0.86 versus 1.74 ± 0.12, p = 4.89 × 10^−8^) (**Fig 2B**). However, dietary fiber intake was significantly lower for the Orang Asli (p < 0.0001) (**Fig 2B**), largely because the Orang Asli participants enrolled in this study were mostly engaged in traditional fish rearing activities in their respective villages and as such, had a high intake of protein from fish. Hence, their diet is quite unlike other rural and indigenous groups that have been characterized with high fiber dietary intake [33]. Principal Component Analysis (PCA) of the combined set of nutritional variables surveyed showed that the overall dietary profiles were clearly distinct between the Orang Asli and urban participants, with macronutrients such as carbohydrates, different forms of lipids (monosaturated and unsaturated fat) and protein (lean meat) being the major distinguishing variables (**Fig 2C**).

Differences in lifestyle can translate into measurable differences in clinical variables in the blood. When we performed PCA on the combined set of blood variables measured through the different clinical panels, we saw that the Orang Asli and urban participants separated into two largely distinct clusters characterized by specific metabolic profiles **(Fig 2D)**. Differences in lipid profiles (such as HDL, LDL, cholesterol and triglycerides), as well as differences in specific liver function variables (such as albumin to globulin (A/G) ratio and the liver enzyme gamma-glutamyl transferase) indicate that there could be differences in the physiology of protein and lipid metabolism between the Orang Asli and urban participants, which could be due to differences in nutritional intake, microbiota or other unknown factors. Hence, Orang Asli and urban participants differ considerably for both nutritional intake and clinical blood variables.

### Variations in gut microbial profiles that are explained by diet, helminths and blood variables

**Fig 3:**
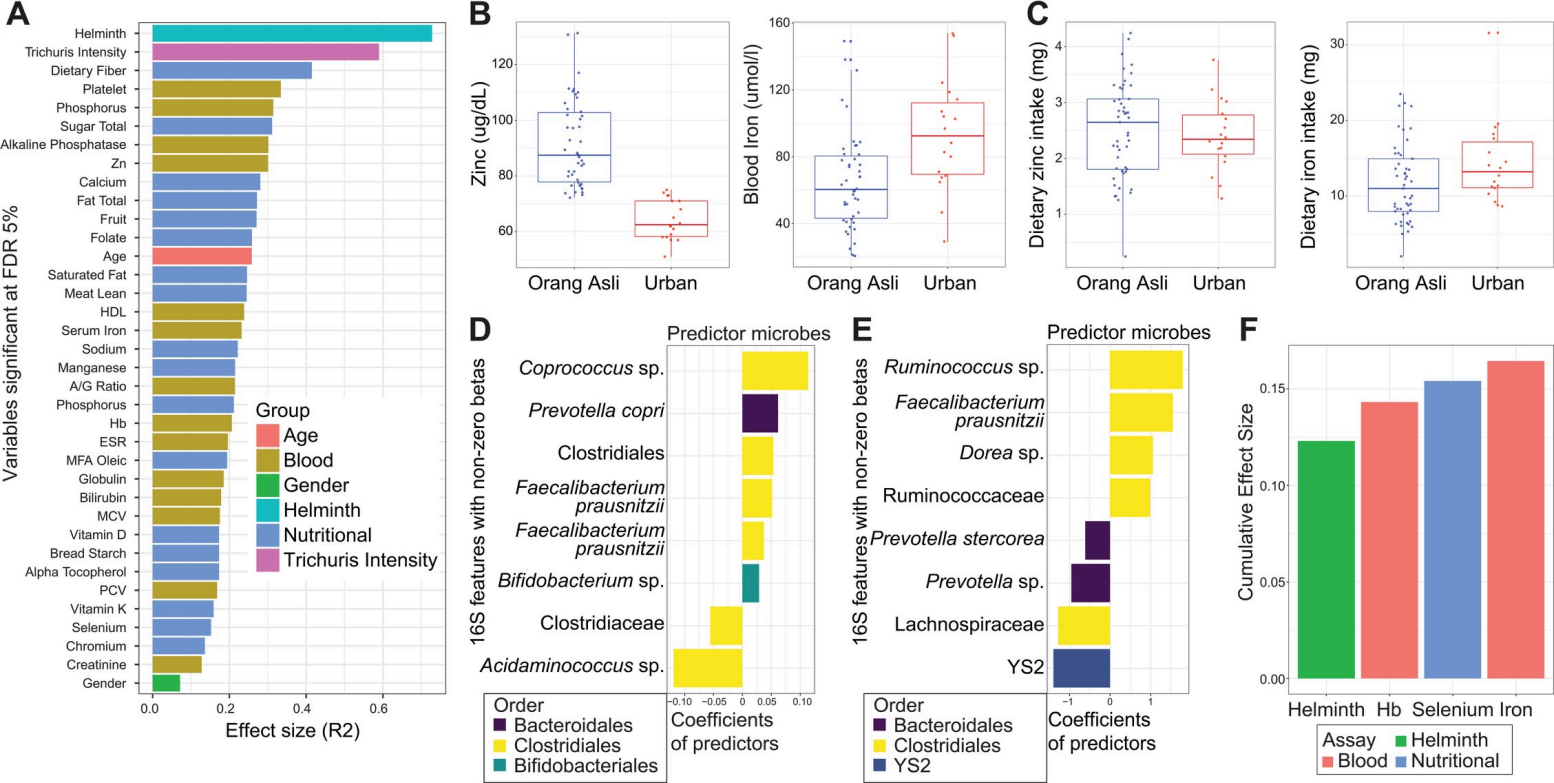
Factors associated with gut microbiota variations. (A) Nutritional, blood and demographic covariates significantly correlated (FDR 5%) to microbiota variation, based on Bray-Curtis dissimilarity of 16S microbial profiles from 40 Orang Asli participants and 18 urban participants. (B) Serum zinc is significantly higher (Mann-Whitney test, p = 1.6 × 10^−9^) among Orang Asli participants (91.6 ug/dL ± 2.3) than among urban participants (64.3 ug/dL ± 1.7). Serum iron, conversely, is significantly lower (Mann-Whitney test, p = 0.002) among Orang Asli participants (65.6 umol/L ± 4.3) than among urban participants (93.0 umol/L ± 7.8). (C) Nutritional daily intake of zinc was similar among Orang Asli participants (2.51 mg/day ± 0.12) and urban participants (2.41 mg/day ± 0.15). Nutritional daily intake of iron trended lower (two-sample t-test, p = 0.07) among Orang Asli participants (11.7 ± 0.7) and urban participants (14.4 ± 1.3). P-values are computed using the two-sample t-test when data is normally distributed; otherwise, the Mann-Whitney test is used. Normality of data distribution is based on the Shapiro-Wilk test of normality using a p-value cutoff of 0.1. Hinges of boxplots correspond to values of the 25^th^, 50^th^ and 75^th^ percentiles, while boxplot whiskers extend to no more than 1.5 × inter-quartile range, beyond which the outlier data points will be plotted individually. (D) Microbial OTUs associated with (D) dietary fiber and (E) *T*. *trichiura* egg burden. Values are coefficients from the SPLS-regression models. OTUs are labeled by taxonomic assignment to the finest possible resolution and color coded by taxonomic order. Repeated taxa names represent two distinct OTUs with the same taxonomic assignment. (F) Non-redundant covariates of microbiota composition, further selected from the 36 significant covariates in (A) based on forward stepwise selection.

We previously demonstrated that Orang Asli and urban participants had different microbiota profiles (**S1 Fig**) [31]. To build upon the additional nutritional and blood variable measurements in this study, we next determined the relative association of these heterogeneous biological variables with the variation of the gut microbial communities between the Orang Asli and urban participants (**S2 Fig**). To reduce the rate of false discovery, we first removed variables with near-zero variance, as well as highly collinear variables (absolute Pearson correlation >0.9). This resulted in 93 metadata variables, which included 47 dietary intake variables, 42 clinical blood variables, overall helminth infection status, *T*. *trichiura* egg count and demographic information (such as age-group and gender). We fit each of these variables to the overall microbiota variation (Bray-Curtis dissimilarity) and identified a set of 36 covariates significantly correlated (FDR 5%) to microbiota variation (**Fig 3A**). Overall helminth infections status, *T*. *trichiura* infection intensity (egg burden) and dietary fiber intake were the top three microbiota covariates, with helminth infections having a relatively higher contribution to gut microbiota variation than diet. In addition to helminth infections and diet, several circulating blood components were also significantly associated with microbiota variation, including metals such as zinc and iron, which have recently been demonstrated to be important in determining disease pathogenesis of many bacterial infections due to their roles as key nutrients for bacteria [34–36]. Serum zinc levels were significantly higher in the Orang Asli participants (65.6 ± 4.3 versus 93.0 ± 7.8, Mann-Whitney test, p = 1.67 x 10^−9^), while serum iron levels were significantly higher in the urban participants (Mann-Whitney test, p = 0.002) (**Fig 3B**). We examined nutritional intake levels of these metals based on the food questionnaire and while dietary iron levels trended higher among the urban participants (14.4 ± 1.3 versus 11.7 ± 0.7 mg/day, Mann-Whitney test; p = 0.051), there was no difference in dietary zinc intake (2.41 ± 0.15 versus 2.51 ± 0.12 mg/day, two-sample t-test; p = 0.61) (**Fig 3C**), indicating that the differences in circulating zinc and iron levels are unlikely due to differences in dietary intake.

We next sought to identify which microbes could have contributed to the associations between dietary fiber, helminth infection and microbiota variation. We focused on dietary fiber and helminth infection as the two covariates most strongly correlated with microbiota variation **(Fig 3A)**. We used centered log-ratio (CLR) [37] combined with Sparse Partial Least Square (SPLS) regression to identify the minimal set of microbes that are associated with either *T*. *trichiura* egg count or dietary fiber intake level pre-treatment **(S3 Fig)**. To minimize the confounding effects between helminth infection status, diet and the two groups of participants, we limited this analysis to only samples from the Orang Asli. We identified six microbial OTUs to be positively associated with dietary fiber levels and two microbial OTUs to be negatively associated with dietary fiber levels (**Fig 3D).** These associations may only apply in contexts such as this one, where the diet is low in fiber on average. We also identified four microbial OTUs that were positively associated with *T*. *trichiura* egg burden, as well as four microbial OTUs that were negatively associated with *T*. *trichiura* egg burden (**Fig 3E**). While many of these selected microbial OTUs from the two different regression analyses summarized in Fig 3D and 3E belonged to the Clostridiales order (11 out of the total 16 selected OTUs), there were differences in the specific genera associated with these two different host variables. For example, *Coprococcus* was uniquely associated with dietary fiber intake, while the genera *Ruminococcus* and *Dorea* were uniquely associated with pre-deworming *T*. *trichiura* egg burden.

We next used a different model selection step to better identify non-redundant microbiota covariates. We performed a forward stepwise selection procedure to identify the minimal combination of the 36 selected variables that would best fit with the overall microbiota variation [38]. This analysis also verified that overall helminth infection status is the most important variable associated with microbiota variation (**Fig 3F**). The next variable that further contributed towards explaining microbiota variation was hemoglobin levels. This was consistent with a previous finding from the Flemish Gut Flora Project (FGFP) where red blood cell count, as well as hemoglobin concentration, were non-redundant microbiota covariates [39]. Dietary selenium intake and serum iron levels were two additional non-redundant covariates identified to contribute towards microbiota variation. Thus, we were able to identify that a combination of covariates, which included intestinal helminth infections, dietary habits and circulating blood components, were associated with variation in the gut microbial communities, with intestinal helminth colonization as the most important microbiota covariate.

### Variations in blood transcriptional profiles that are explained by diet, helminths and blood variables

**Fig 4:**
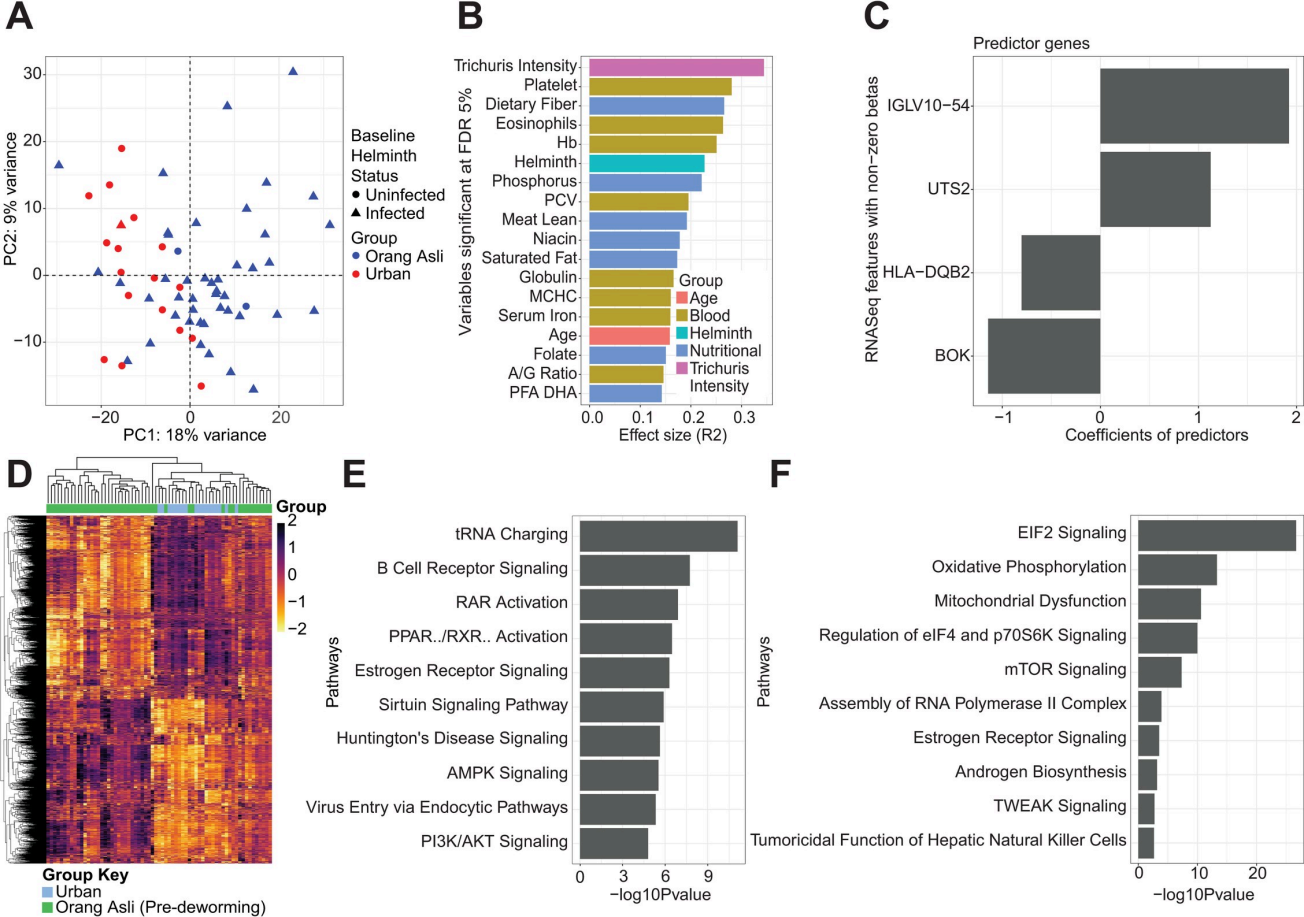
Transcriptional signatures associated with *T*. *trichiura* egg burden. (A) PCA plot based on transcriptional profiles of 46 Orang Asli and 18 urban participants, with individuals infected and uninfected with helminths denoted by circles and triangles, respectively. Only transcriptional profiles with matching nutritional and blood profiles were included in this analysis. (B) Nutritional, blood and demographic covariates significantly correlated (FDR 5%) to variation in transcriptional profiles. (C) Genes associated with *T*. *trichiura* egg burden and the associated coefficient values from the SPLS-regression model. (D) 4636 genes identified with differential expression in 18 urban participants vs. 49 Orang Asli participants at FDR 10%. Pathways enriched in (E) 2188 genes with significantly higher expression levels among urban participants and (F) 2448 genes with significantly higher expression levels among Orang Asli participants.

To produce a more comprehensive assessment of the host response in the context of intestinal parasite colonization, we performed RNA-seq on PAXgene samples and examined genome-wide transcriptional profiles of peripheral whole blood from Orang Asli and urban participants **(S4 Fig)**. Based on PCA, Orang Asli participants prior to being dewormed and urban participants had distinct transcriptional profiles overall (**Fig 4A).** We then applied the same approach as described above for the microbiota towards identifying variables that have the greatest effect size on blood transcriptional variation. Similar to the variation in gut microbiota, *T*. *trichiura* egg count was also a top covariate significantly associated (FDR 5%) to the variation in blood transcriptional profiles (**Fig 4B**). However, the overall helminth infection status was less associated with variations in blood transcriptional profiles. Blood eosinophil abundance was also identified as a covariate of blood transcriptional profiles, in line with the roles of eosinophils in mediating the outcomes of helminth infections, best demonstrated in various mouse models of infection [40].

Similar to identifying OTUs from microbial 16S data that were associated with host covariates, we next used SPLS-regression to identify genes associated with *T*. *trichiura* egg counts among the Orang Asli participants pre-deworming and differences in the expression levels of these genes should be associated with *T*. *trichiura* burden. We found genes *IGLV10-54* (the lambda light chain of immunoglobulin variable 10–52) and *UTS2* (urotensin 2) to be positively associated with egg counts, while *HLA-DQB2* (part of the human class II major histocompatibility complex) and *BOK* (BCL2 family apoptosis regulator) were negatively associated with egg counts (**Fig 4C**). The identification of *HLA-DQB2* and *IGLV10-54* as predictor genes of pre-deworming egg count suggests that differences in specific aspects of the immune system, such as antigen presenting capability and humoral adaptive immunity, could be associated with the underlying cellular mechanisms that contribute to population variability in *T*. *trichiura* egg burden.

We next took a supervised approach to identify transcriptional profiles that distinguished between the Orang Asli and the urban participants. 4636 genes were differentially expressed; 2188 and 2448 genes were upregulated in the urban participants and in the Orang Asli, respectively (**Fig 4D**). By pathway enrichments analysis, the genes more highly expressed in urban participants were enriched for B cell receptor signaling pathway (**Fig 4E**). The genes with significantly higher expression in Orang Asli participants were enriched for the EIF signaling pathways (stress-response signaling pathways activated during bacterial infections) [41] and this could be due to the more frequent exposure to infectious microorganisms among the Orang Asli (**Fig 4F**).

### Changes in blood transcriptional profiles caused by deworming

**Fig 5:**
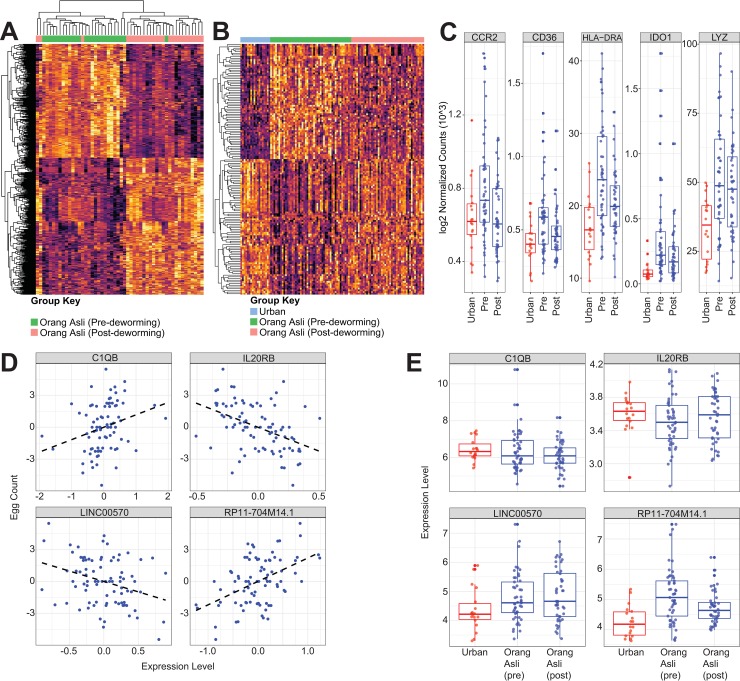
Transcriptional signatures associated with changes post-deworming. (A) 654 genes with significantly altered expression post-deworming (FDR 10%) among 42 Orang Asli participants. (B) Heatmap showing expression levels of 148 genes that were likely helminth-dependent and (C) boxplots highlighting the expression patterns for five of these genes with a role in helminth immunity. (D) Regression of transcriptional profile against change in *Trichuris* burden with deworming. (E) Predictive genes from regression in (D) with expression in urban participants included.

The longitudinal component of this study enabled us to identify changes in the blood transcriptional profiles directly caused by deworming. In order to detect genes with altered expression due to the reduction or absence of *T*. *trichiura*, we identified 26 individuals who responded to the deworming treatment (defined as individuals who had at least a 2-fold reduction in *T*. *trichiura* burden post-deworming treatment) and performed paired comparisons, where the post-deworming transcriptional profile from each individual is compared to the individual’s pre-deworming blood transcriptional profile. We identified 654 genes with significantly altered expression levels (FDR <10%) after deworming treatment (Fig 5A). Of this list of significant genes, we further identified a subset of 148 genes by comparison with data from the urban controls– 80 of them had higher expression levels pre-deworming and higher expression in Orang Asli as compared to urban controls, while 68 of them had higher expression levels in both post-deworming samples in the Orang Asli and urban participants (Fig 5B). Hence, this union set of 148 genes likely represent genes that were related to helminth infection. 29 of these 148 genes were associated with immune system processes, as defined using the gene ontology term “Immune System Process” (gene ontology identifier: 0002376) (**S5 Fig, S4 Table**), including five genes (*CCR2*, *CD36*, *HLA-DR*, *IDO1*, *LYZ*) that are well known to be expressed by myeloid cells (**Fig 5C**).

We also performed SPLS-regression using changes in *T*. *trichiura* burden post-deworming as a dependent variable, to identify specific genes that are directly related to changes in *T*. *trichiura* burden. Notably, a reduction in *T*. *trichiura* burden was associated with an increased expression of *IL20RB* **(Fig 5D)**. While *IL20RB* had higher expression among pre-deworming Orang Asli participants when compared to urban participants, its expression level among Orang Asli participants was reduced to resemble that in urban participants after deworming treatment **(Fig 5E)**.

**Fig 6:**
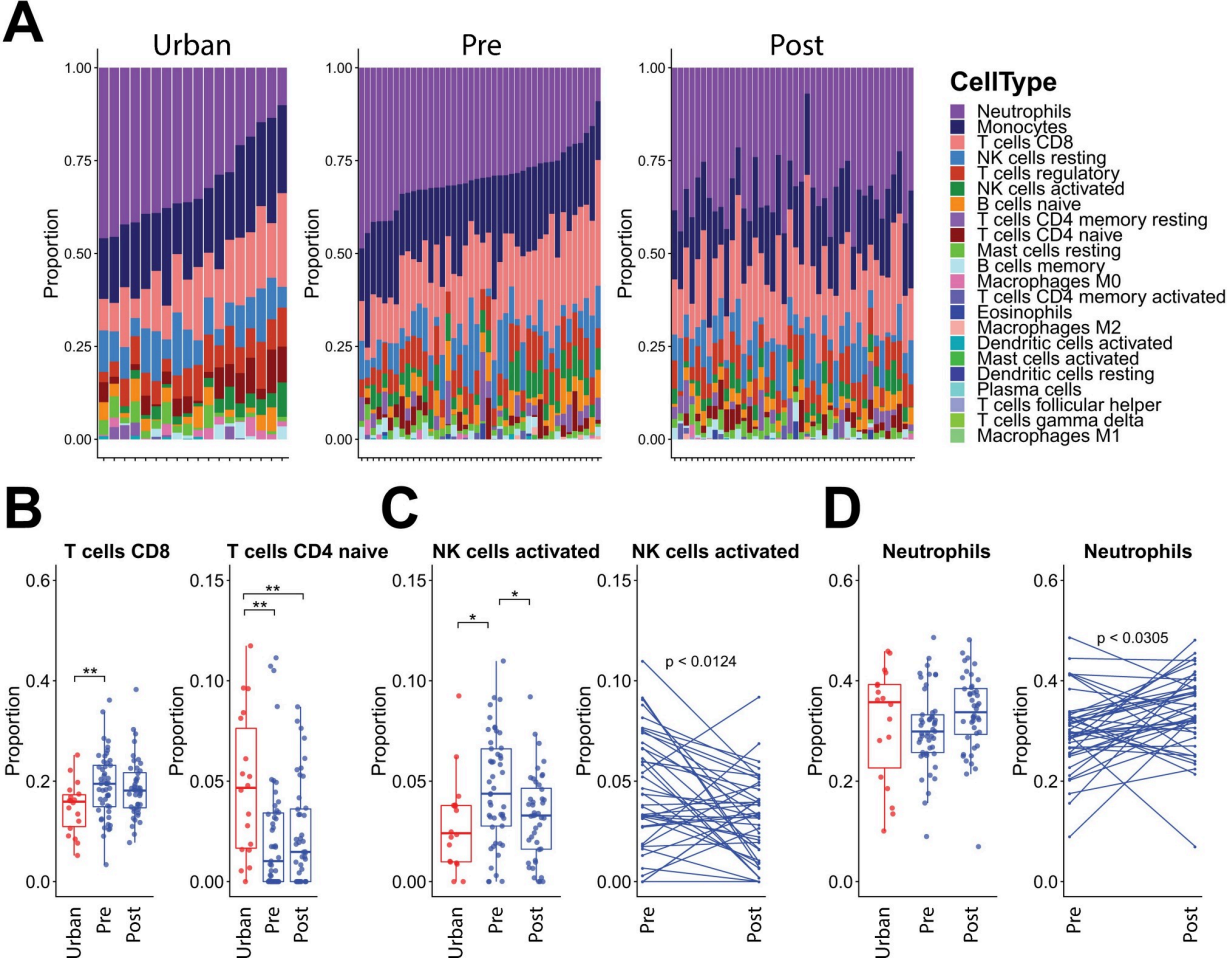
Immune cell type estimates from blood transcriptional profiles. (A) Immune cell type proportion estimates from default CIBERSORT [74] deconvolution of 22 cell types in urban, Orang Asli pre- and post-deworming blood transcriptional profiles. Matched samples from Orang Asli pre- and post-deworming are shown in the same order along the horizontal axes. (B) Significant differences between urban and Orang Asli, and before or after deworming, samples are shown for CD8+ T cells, naïve CD4+ T cells, (C) monocytes and (D) activated NK cells according to bootstrapped one-way ANOVA with 999 simulations and subsequent Tukey with multiple test correction. * = p≤0.05; ** = p≤0.01; *** = p≤0.001. Additional Wilcoxon tests indicate significant differences between matched villager before and after samples for (C) monocytes and (D) activated NK cells.

While flow cytometry analysis was not performed to assess immune cell populations in the blood, recent algorithms have been developed to infer abundances of different cell types in a mixed cell population with transcriptional profiling data [74]. Based on this CIBERSORT analysis (**Fig 6A**), the Orang Asli were inferred to have significantly higher abundance of CD8+ T cells, but fewer naïve CD4+ T cells, as compared to the urban participants (**Fig 6B, S5 Table**). The differences in the proportions of these T lymphocytes did not appear to be altered by deworming, thereby suggesting that these could represent differences in immunity that are helminth-independent. In contrast, monocyte proportions decreased significantly post-deworming, but were similar between urban and Orang Asli participants pre-deworming (**Fig 6C**). The Orang Asli were predicted to have a significantly larger population of circulating activated NK cells that were also reduced after deworming treatment (**Fig 6D**), consistent with reports of NK cell activation by helminths [42–44]. We examined whether the change in the proportion of cells made up by each of these four cell populations was correlated with the change in egg burden post-deworming (**S6 Fig**). No significant relationships were seen. This could mean that post-deworming changes resulted from something other than the change in *Trichuris* burden.

### Associations between fecal microbial communities and blood transcriptional profiles

**Fig 7:**
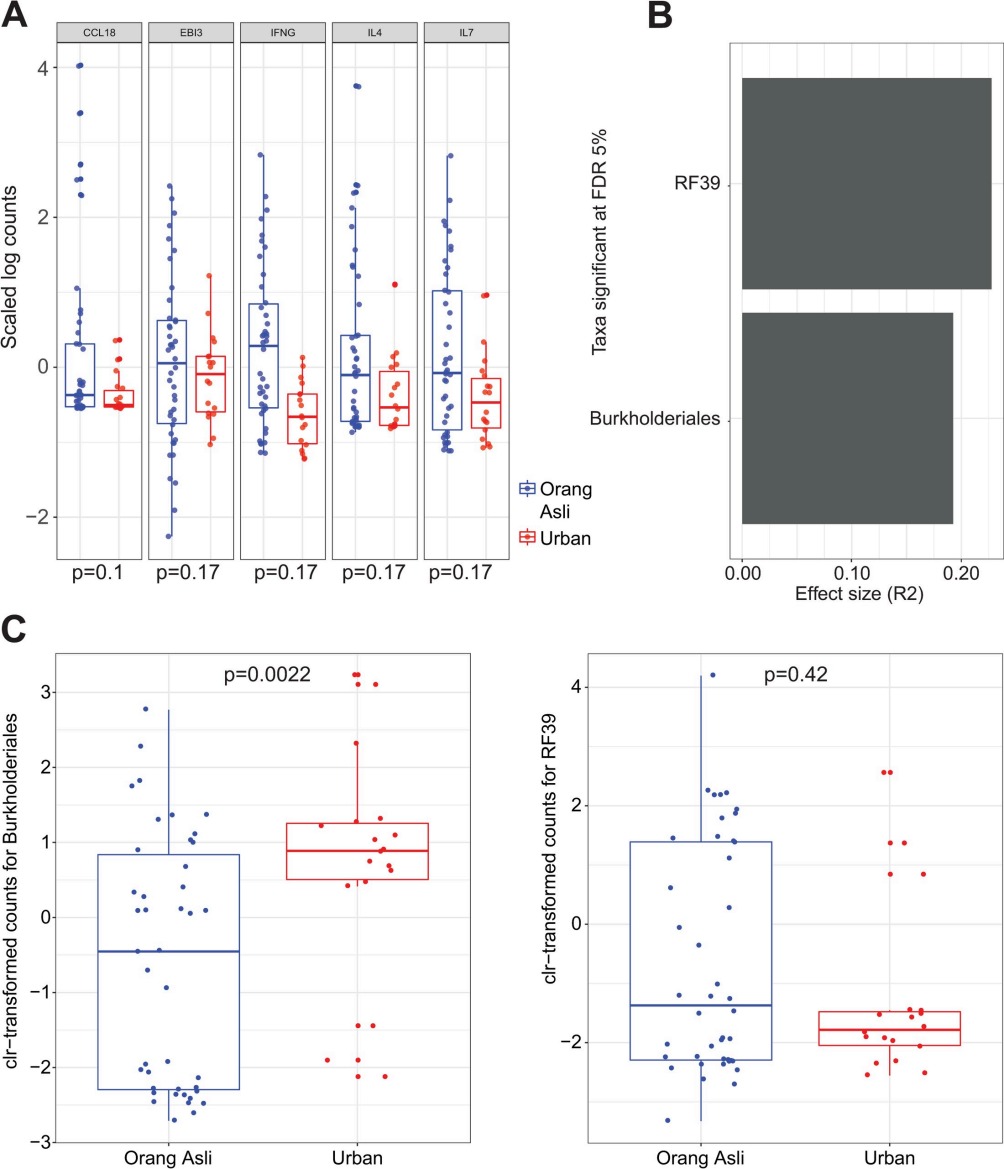
Microbial communities and blood transcriptional profiles. (A) Genes with significant differences in variance between Orang Asli and urban participants. P-value was based on the Fligner-Killeen test and adjusted by the Benjamini-Hochberg procedure for the multiple immune response genes tested. Adjusted p-values are visualized. (B) Taxa identified as significantly associated with variation in blood immune gene expression levels. (C) Comparison of CLR-transformed counts of the two taxa identified in (D) between Orang Asli and urban participants. P-values were based on Mann-Whitney tests and the normality of the data distribution was determined by the Shapiro-Wilk normality test using a p-value cutoff of 0.1.

Gut microbiota has been linked to population variation in cytokine production by circulating blood monocytes in a cohort of 500 healthy Western-European individuals [45]. Adopting a similar approach, we next identified significant associations between microbial features and specific immune response genes among the Orang Asli and urban participants. We first analyzed the inter-individual variability in the expression of genes annotated as chemokines and cytokines (a union set of 82 chemokine and cytokine genes) as a representation of immune response genes. Variance of the expression level of chemokine and cytokine genes in the blood was similar between the Orang Asli and the urban population (Fligner-Killeen test, p = 0.11) (**S7 Fig**). Of the 82 cytokine and chemokine genes, the genes with the biggest difference in variance between the Orang Asli participants and urban participants were *IFNg* (adjusted p-value = 0.1), *CCL18*, *IL-7*, *EBI3* and *IL-4* **(Fig 7A)**.

We next quantified how much of the overall variation of each transcript could be attributed to the gut microbiota. The microbial abundance data was summarized at 30 taxonomic order levels and fit to the variation in cytokine and chemokine profiles. We found the orders Burkholderiales and RF39 to be significantly correlated with variation in circulating chemokine and cytokine gene expression (**Fig 7B**). Burkholderiales was significantly more abundant in the urban participants (Mann-Whitney test, p = 0.0022). The mean abundance of RF39 was somewhat (but not significantly) higher in the Orang Asli participants (Mann-Whitney test, p = 0.42) (**Fig 7C**).

### Helminth dependent covariates of microbial and blood transcriptional profiles

**Fig 8:**
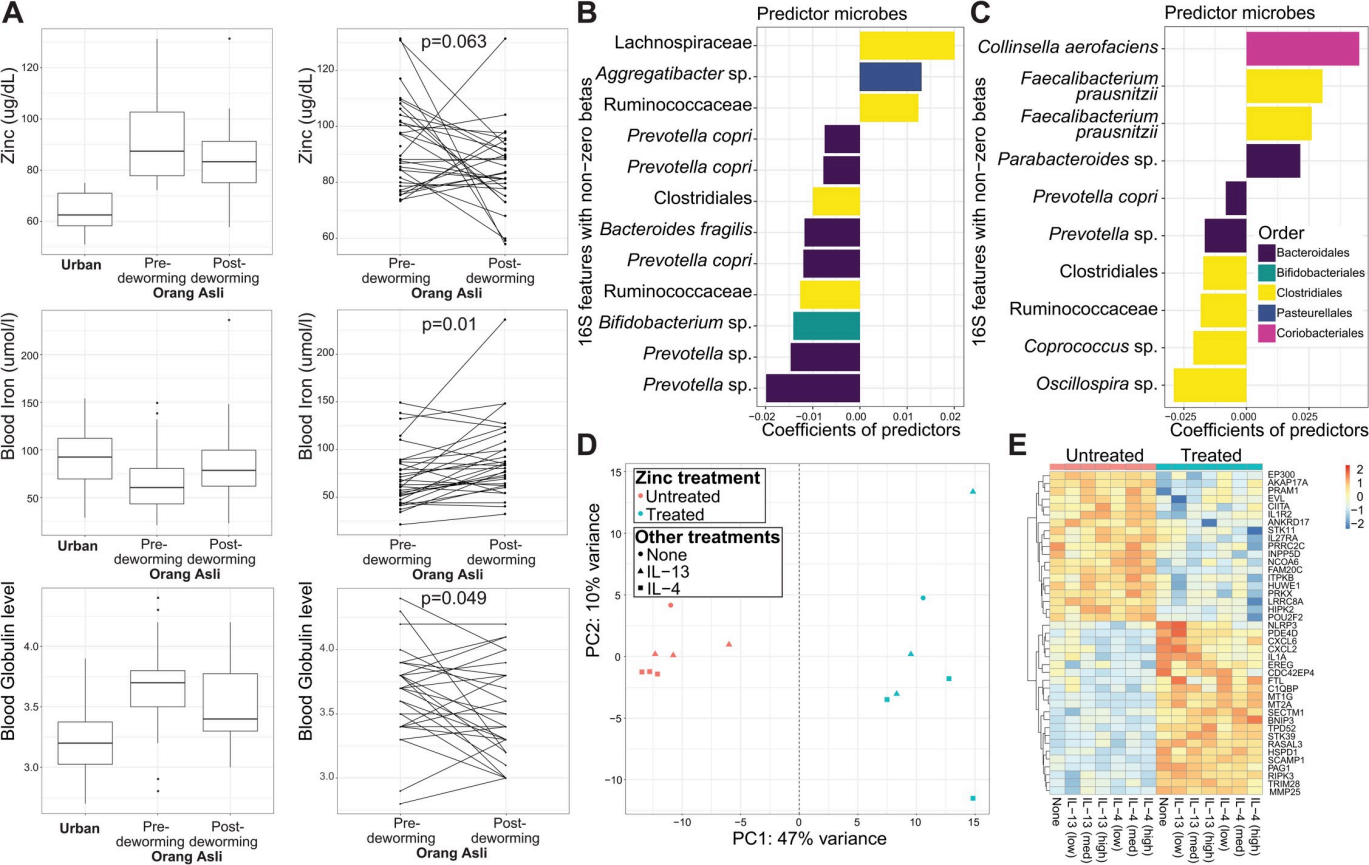
Helminth dependent covariates of microbial and blood profiles. (A) Blood covariates that responded significantly to deworming, with changes in values that resembled values seen in urban participants, as demonstrated using the multi-group boxplots (left). Values for boxplots are based on 18 urban participants and 46 Orang Asli participants with pre-deworming serum chemistry values, as well as 34 Orang Asli who received deworming treatment and had serum iron and serum globulin levels measured post-deworming and 33 Orang Asli who received deworming treatment and had serum zinc levels measured post-deworming. (Right) Connected line plots for paired comparisons of 30 pairs of pre- and post-deworming samples for serum zinc levels, as well as 31 pairs of pre- and post-deworming samples for serum iron and globulin levels. P-values for two-group comparisons (pre-deworming Orang Asli vs. urban participants) are based on t-tests (when data is normally distributed) and Mann-Whitney tests (when data is not normally distributed). Normality of data distribution is determined using the Shapiro-Wilk test of normality. Statistical significance of deworming changes are based on paired t-test of pre- and post-deworming paired values. Microbial OTUs associated with changes in (B) serum zinc levels and (C) serum iron levels post-deworming treatment are shown; values are coefficients from SPLS-regression models. Selected microbial OTUs are labeled with taxonomic assignments of the finest possible resolution and color coded by taxonomic order. Repeated taxa names represent two distinct OTUs with the same taxonomic assignment. (D) A PCA plot based on the 1835 most variable genes (85^th^ percentile) shows separation between transcriptional profiles of macrophages that were treated (teal) or untreated (pink) with zinc sulfate. Some samples were treated with IL-4 (squares) and IL-13 (triangles) as well. (E) Heatmap of the 41 immune-related genes (GO:0002376) that are significantly differentially expressed in zinc-stimulated versus unstimulated experiments (cutoff p = 0.01, |log2 fold-change| > 1).

We took advantage of the deworming component of our study to identify which of the gut microbiota and blood transcriptional profile covariates measured from the clinical blood panels were helminth-dependent. We focused on the subset of blood covariates with repeated measures in the Orang Asli participants pre- and post-deworming (HDL, globulin, zinc, iron and total bilirubin) to determine which of these covariates were significantly altered by deworming to resemble the levels in urban participants and therefore, likely to be helminth-dependent. We found serum zinc, iron and globulin levels to match these criteria (paired t-tests, adjusted p-values < 0.01), although the directions of change were different (**Fig 8A**). Changes in serum zinc levels were positively correlated with changes in *T*. *trichiura* egg burden (Pearson correlation = 0.51, p = 8 × 10^−5^) and changes in serum iron were negatively correlated with changes in egg burden (Pearson correlation = -0.28, p = 0.033), indicating that changes in serum zinc and iron levels are related to *T*. *trichiura* infection.

Metal metabolism both in the mammalian host and bacteria can determine the outcome of a bacterial infection [34, 36, 46]. Metals are important nutrients to bacteria and the mammalian hosts have evolved specific cellular strategies to restrict acquisition of metals by bacteria [35]. To determine if changes to the circulating levels of zinc and iron could be correlated with specific changes in microbial communities, we utilized CLR transformation with SPLS-regression on the pre- and post-deworming samples to identify specific microbial OTUs that correlated with changes in serum zinc and iron levels, respectively (**S8 Fig**). We identified 12 microbial OTUs that were associated with changes in circulating zinc levels. The majority of microbial OTUs with positive associations (2 out of 3 microbial OTUs) belonged to the Clostridiales order and the majority of the microbial OTUs with negative associations (6 out of 9) belonged to the Bacteroidales order (**Fig 8B**). Changes in circulating iron levels were associated with 10 microbial OTUs, predominantly from the Clostridiales order in both directions of association (**Fig 8C**).

In order to probe the significance of increased levels of zinc in the blood from pre-deworming OA individuals, we examined the transcriptional response to zinc of macrophages *in vitro* by RNA-seq. We stimulated human monocyte-derived macrophages with zinc sulfate, in combination with 3 different concentrations of IL-4 or IL-13 (cytokines associated with a Th2 response to helminth infection). When the transcriptional profiles were visualized by PCA, samples stimulated with zinc clearly separate along PC1 from those unexposed to zinc (**Fig 8D**). The top ten genes contributing to PC1 were all metallothioneins (**S9A Fig**). Metallothioneins bind zinc and copper, as well as other metals, and are involved in their regulation. Metallothioneins are associated with both an increase in intracellular zinc and in bacterial clearance [47]. Some immune-related genes, such as CXCL6 and IL-1B were also found among the top 50 genes contributing to PC1 (**S9A–S9B Fig**). We then looked specifically at immune-related genes (GO:0002376) that were differentially expressed in macrophages exposed to zinc, shown in **Fig 8E**. Upregulated genes include NLRP3, CXCL6, CXCL2 and C1Q binding protein. These results suggest that perhaps increased serum zinc levels could combine with type-2 cytokines to boost anti-bacterial responses in human macrophages.

Hence, we identified specific microbial taxa and host transcripts that are associated with changes in circulating zinc and iron levels, as a result of deworming. While the biological implications of these associations would require further experimentation, these results identify previously unexpected links between circulating zinc and iron levels, with helminth infection status, gut microbial communities and the activation state of peripheral immune cells.

## Discussion

In this study, we generated a comprehensive profile of blood variables, as well as dietary intake data from helminth-infected individuals living in a rural setting in Malaysia along with urban participants. The goal was to identify the important factors influencing the gut microbiota and to determine the relative impact of helminth infection on host responses and the microbiota. Although the number of individuals studied here is relatively small, the comprehensive nature of this dataset also enabled us to identify new factors (e.g. serum zinc and iron levels) that could be modulated by helminth infections and their associated microbial communities, which might have important associations with the host immune response.

Our analysis indicates that helminth infection is indeed one of the most important variables that can explain inter-individual variation in microbial communities. While diet is a well-established source of variation in microbial community abundance, the impact of helminth infection has a relatively larger effect size on microbial variation than dietary fiber intake (R^2^ values of 0.73 vs. 0.41, respectively), which constitutes an important conclusion from our data. It is also important to note that the most prevalent worm in the Orang Asli who participated in this study is *T*. *trichiura—*the whipworm that colonizes the large intestine. Any changes to fecal microbial communities effected by worms that reside in the small intestine (such as hookworm and *Ascaris*) might be too attenuated during subsequent passage through the large intestine to be detected in excreted stool. Interestingly, while the overall presence of helminth in the gut accounted for less of the inter-individual variation in blood transcriptional profiles, pre-deworming *T*. *trichiura* egg burden still remained the top covariate of blood transcriptional profiles, suggesting that the helminth-associated blood transcriptional profiles could be specific to the worm species.

The other major observation that is of interest for future studies is the relationship between circulating levels of transition metals (zinc and iron) and inter-individual variations in gut microbiota, as well as blood transcriptional profiles. The availability of these transition metals shapes the pathogenesis of infections and this concept is largely termed “nutritional immunity” [35]. For example, increased dietary zinc intake in mice leads to increased susceptibility to *Clostridium difficile* infection (CDI), and exacerbated CDI-associated disease. However, in contrast to the mouse study where mice were fed with increased dietary zinc, the differences in circulating zinc and iron levels in our study are not results of differences in dietary intake and as such, are more likely to be a result of other factors that should be investigated. The complex relationship between zinc and immune system is evident from the finding that expression of metallothioneins leads to increased intracellular zinc and clearance of bacteria in macrophages [47]. When we stimulated macrophages *in vitro* with zinc, in the presence of IL-4 and IL-13, we found that several metal-metabolism genes, as well as several immune-related genes, were upregulated. Possible factors include the direct effect of helminths in the gut, alterations to metal metabolism pathways utilized by the worm-associated gut microbial communities or differences in the host genetics regulating metal homeostasis. While to our knowledge no study participants were suffering from *Trichuris* dysentery syndrome, this condition has been associated with iron-deficiency anemia [48]. In addition, there may be implications of having different levels of circulating metals on the blood immune cells. Zinc has previously been shown to induce a tolerogenic phenotype in dendritic cells by suppressing the expression of *MHCII*, promoting expression of *PD-L1*, *PD-L2*, and *IDO* [49], as well as inhibiting the signaling pathways of lambda interferons [50].

Furthermore, several large cross-sectional population studies have reported association between serum zinc levels and helminth infections, although the direction of association was not always consistent. Three of these studies reported an inverse relationship–Kongsbak *et al*. reported that *T*. *trichiura* was associated with low serum zinc [51], while Arinola *et al*. and Akinwande *et al*. independently reported that *Ascaris lumbricoides* infection was associated with low serum zinc in children and pregnant women [52, 53]. In contrast, a study in Vietnam found that zinc deficiency was significantly less common only among children with single infection of *T*. *trichiura* only [54]. However, the same group also reported the lack of significant relationship between serum zinc levels and helminth infection among children in Cambodia [55]. It should be noted that these studies were mostly done among children, while our study recruited participants across all ages (age of participants ranged from 6 to 58 years) and therefore, is considerably more variable in terms of demographics. In addition, the most prevalent gut helminth infection in our study is *T*. *trichiura*, followed by hookworm, while the studies described here were largely conducted in regions where *Ascaris* was more prevalent. In fact, the study among Nigerian children by Akinwande *et al*. reported that *T*. *trichiura* was least prevalent and where children were infected only with *T*. *trichiura* in Vietnam, they had significantly lower prevalence of zinc deficiency, which is comparable to our findings. The differences in these findings also suggest that the interplay between gut helminth infection and circulating micronutrients (such as zinc) are more likely to be indirectly mediated by additional factors, such as differences in baseline gut microbiota and host genetic variations.

A major caveat in our study is that most variables are strongly associated with membership in either the urban or the Orang Asli cohort. For example, helminth infection occurs almost exclusively among Orang Asli subjects, while only one of the urban subjects was infected. The distribution of dietary fiber intake among Orang Asli and that among urban subjects were almost non-overlapping (Fig 2B). Children participants were only recruited among the Orang Asli group, but not among the urban group (**S10 Fig**). Consequently, these different variables confound each other. While this makes it difficult to separate out the effects of helminth vs. dietary fiber on the gut microbiota, when ranked by degree of association, helminth infection still has a relatively higher contribution to microbiota differences. This conclusion is also reinforced by the use of a different model selection method (Fig 3F), which shows that by selecting for only non-redundant variables, helminth infection is still a significant covariate associated with microbiota differences (but not dietary fiber). While albendazole treatment may have had some effect on the microbiome, we believe that this is likely limited. Recent studies in Indonesia and Kenya found statistically significant but small changes in the microbiome that could have been a direct result of albendazole treatment [56, 57]. Since prevalence in our study site was so high (96%) that we did not have an uninfected post-deworming control group, we believe the strongest evidence of changes resulting from worm clearance are those where changes are correlated with the decrease in egg burden (**Fig 4**) and those in which the post-deworming phenotype reverts to a more urban-like phenotype (**Fig 5**).

Overall, by examining heterogeneous data sets with perturbation (deworming) and repeated measures, we have been able to infer several helminth-associated and helminth-dependent biological interactions that present future directions for more in-depth mechanistic and experimental studies. In addition to mechanistic studies, current efforts are also underway to establish similar field studies that are of larger scale to determine if these inferred biological interactions can be reproduced in a different and larger cohort. Furthermore, a larger longitudinal cohort will also allow us to examine additional questions and to address flaws of the current study. This includes the ability to stratify study participants into finer categories of deworming treatment-resistant and treatment-responsive individuals, to control for helminth-independent factors that could also contribute to population variation in gut microbiota and blood transcriptional profiles (which could not be done in this current study due to the high prevalence of helminth infection and lack of helminth-negative individuals among the Orang Asli community), as well as to identify host genetic variations that could also account for the inter-individual variations in gut microbiota and blood transcriptional profiles in these indigenous communities.

## Materials and methods

### Ethics statements

The study was approved by the Research and Ethics Committee of the University Malaya Medical Centre (UMMC) Malaysia (i.e., MEC Ref. No. 824.11 and No. 943.14). In addition, permission was also obtained from the Department of Orang Asli Development (JAKOA) and the Tok Batin (chieftain) of the respective village before the study was initiated. Consenting villagers participated in the study after a clear explanation of the study objectives and written consent were given. Parents signed on behalf of their children who were younger than 12 years of age. The villagers were also assured that their personal information will be kept confidential and they have the right to withdraw from the study anytime without indicating any reasons. Researchers were not blinded to the different groups. Study inclusion criteria consisted of membership in the Temuan sub-tribe and residency in the target village. Exclusion criteria were (1) being pregnant or possibly pregnant (self-reported); (2) presence of diagnosed or perceived chronic diseases; (3) recent history of anthelminthic treatment and antibiotics (within 6 months of the start of the study) and (4) having a fever.

### Study design and sample collection

For this study, the Temuan subtribe from a village situated in Hulu Langat district in the state of Selangor, was selected because of ease of access to the location, as well as good rapport with the researchers (S.C.L., Y.A.L.L.). Approval was given by the local health authorities for treatment activity to be carried out in this village. This study was designed with the goal to evaluate helminth-dependent and helminth-independent effects on stool and blood profiles. Therefore, the intestinal helminth infection and cure rates at 21 days after deworming treatment were examined. Light microscopy was used to detect helminth eggs, as well as other intestinal protozoan parasites. *Entamoeba histolytica* and *Giardia lamblia* were found in five samples. Details of the study design, including procedures of fecal samples collection and detection methods for intestinal helminth, are described in Ramanan and Bowcutt [31]. The number of samples positive for protozoan infections by microscopy (n = 5) was insufficient to justify further analysis, except to note that these individuals did not cluster separately from the other Orang Asli samples in terms of similar microbiomes (**S11 Fig**).

Since many study participants remained infected with *Trichuris* post-deworming, we previously classified the response to treatment among the Orang Asli samples by measuring the difference between log-transformed *Trichuris* levels before and after treatment. Response to deworming clustered into two distinct groups based on a threshold of -Δ0.03 (responders and non-responders). We then identified a small set of bacterial OTUs for which a linear combination of abundance changes accurately models concurrent changes in *Trichuris trichuria* egg burden [31].

Besides fecal samples, blood samples were also collected before and after deworming treatment for clinical blood tests and whole blood transcriptional profiling. Briefly, a maximum of 10ml venous blood was drawn from each participant by a phlebotomist into a PAXgene blood RNA tube, a plain tube (collection tube without anticoagulant), an EDTA tube (collection tube with the anticoagulant ethylene diamine tetraacetic chloride) and a sodium fluoride tube. The PAXgene tubes were stored frozen at -80°C until RNA isolation. The remaining tubes were sent to B.P. Clinical Lab Sendirian Berhad (Shah Alam, Selangor, Malaysia) to perform the clinical blood tests. Where matching stool and blood data sets are available, subsets of samples were used to perform comparisons of interest (**S2 Table)**.

### Dietary intake assessment

Dietary intake was inferred using 7-day dietary records. Participants were interviewed face-to-face continuously over 7 days to measure their food intake using pictures of food, as well as different sizes of cups, bowls and spoons. Brand names of food, the method of preparation and cooking, recipes for composite dishes and portions of food were recorded in detail. Parents or guardians answered on behalf of those younger than 12 years old. Subsequently, the nutritional content of foods consumed was calculated and analyzed using Nutritionist Pro (Axxya Systems, Stafford, TX, USA) based on the Malaysian Food Composition Table [58].

### Analysis of blood and nutritional profiles

To reduce potentially redundant variables, we filtered and removed blood and nutritional variables based on variance and pairwise correlation values (**S2 Fig**). We used filtering functions from the R package *caret* [59]. We first removed variables with near-zero variance, followed by removal of highly correlated variables based on the Pearson correlation values. If two variables have high correlation (defined as an absolute Pearson correlation of >0.9), the mean correlation value for each of the variable with all other variables in the data set was determined and the variable with the larger mean correlation value was removed. These filtering steps were implemented for the blood and nutritional profiles separately, resulting in a final set of 47 blood variables and 42 nutritional variables. To perform Principal Component Analysis (PCA), we centered and scaled the values for each of these blood and nutritional variables across samples (z-score transformation to mean of 0 and standard deviation of 1). PCA scores, as well as the variables with the five most positive and negative loading values on PC1, were visualized as two-dimensional biplots using custom scripts from the *ggbiplot* package [60].

### 16S rRNA gene sequencing

DNA was extracted from fecal samples for microbial analysis using the Macherey-Nagel NucleoSpin Soil kit (Macherey-Nagel GmbH & Co. KG, Düren, Germany). The 16S rRNA gene was amplified at the V4 hypervariable region and sequenced according to the multiplexing protocol described by Caporaso *et al*. [61, 62] on the Illumina MiSeq system paired-end sequencing for 2 x 150bp reads.

### 16S rRNA gene sequencing reads processing

16S rRNA sequencing reads were processed using the QIIME2 suite of tools [63]. We first demultiplexed sequencing reads and inspected the read quality scores across all read positions. Since the mean quality score at all read positions were greater than 30, we did not trim sequences in subsequent sequencing read processing steps. We next used DADA2 [64] to define microbial features. We used the term “Operational Taxonomic Units (OTUs)” to describe the microbial features identified by DADA2, since this term has been widely used in 16S rRNA sequencing studies. We next used the Naïve Bayes classifier along with reference sequences from the Greengenes database (reference sequences grouped at 97% similarity) to train a taxonomic classifier, which was subsequently used for taxonomy assignments for the OTUs identified in our study.

### Microbial variation and covariate analysis

We used functions from the R package *vegan* [39, 65] to identify biological covariates associated with variations in microbial profiles (**S2 Fig**). All functions used were implemented with default parameter values, unless stated otherwise. We first removed microbial features with low abundance, defined as OTUs with less than 10 counts in less than 10% of the selected samples. We then calculated the Bray-Curtis dissimilarity for these 16S profiles and performed Principal Coordinate Analysis (PCoA) on this dissimilarity matrix. Variations in the gut microbiota were defined as PCoA scores of the first two components. We compiled the 47 blood variables, 42 nutritional variables, age category (where participants of age 18 years and older were categorized as “Adult”, while participants of age younger than 18 years were categorized as “Children”), gender, overall helminth status and pre-deworming *T*. *trichiura* egg count as a set of 93 biological covariates of interest. To identify which of these 93 biological covariates were significantly correlated with variations in the relative abundance of gut microbiota, we fit the values of each of these variables to the Bray-Curtis dissimilarity PCoA scores of the first two components using the *envfit* function and computed the significance p-value by permutation. To control for multiple hypothesis testing, we used the Benjamini-Hochberg (BH) procedure to compute an adjusted p-value for each fit and defined statistically significant covariates at a false discovery rate (FDR) of 5%. We visualized the 33 significant variables using the goodness-of-fit statistics squared correlation (R^2^) in order of decreasing magnitude.

To further select for a subset of non-redundant covariates from the 33 significant microbiota covariates, we used the function *ordiR2step* to perform forward subset model selection only among the significant covariates. Briefly, this procedure begins by comparing a null model containing no variables (i.e. the first two components of the PCoA scores defined above is fit to the intercept only) and a test model containing one variable, where every possible variable is considered. A p-value computed by permutation is used to determine if the differences in squared correlation values between the null and test models were statistically significant. The function then builds model forward by increasing the number of variables in the model one-at-a-time and will stop once the p-value threshold is exceeded, as this indicates that further addition of any variable does not improve model performance (defined by R^2^) when compared to the null model. We used a p-value cutoff = 0.1 and visualized the R^2^ values with each increment of model size.

### Sparse partial least square regressions (SPLS-regression) of 16S microbial profiles with host covariates

We used a statistical learning framework known as sparse partial least square regression (SPLS-regression) to identify a minimal set of microbial features that were significantly associated with a host covariate (for example, dietary fiber intake, pre-deworming *T*. *trichiura* egg count) while reducing spurious correlations [60, 66]. Briefly, SPLS-regression seeks to identify a narrow (“sparse”) set of independent variables in a subspace with reduced dimensions (“latent components”) significantly associated with the dependent variable of interest. We first removed low abundance OTUs and performed centered log-ratio (clr) transformation on the OTU count data of the filtered OTU matrix to account for the compositional nature of 16S microbial profiles. We next transformed the dependent variables in data-specific manner–dietary fiber intake was log_10_ transformed, while pre-deworming egg count was added with a unit pseudocount followed by log_2_ transformation.

To determine the approximate number of latent components that were of interest, we performed PCA on the clr-transformed OTU matrix to identify the number of principal components that would explain for 30% of the total variance within the data set. We then determined the optimal sparsity parameter by performing model selection using a stability selection approach [31, 67]. In this approach, we built subsampled SPLS-regression models (80% of the sample size and over 20 repeats) over a range of sparsity values and determine the minimum amount of sparsity that would still result in model variability below that of the acceptable threshold. We fit the SPLS-regression model with the selected number of latent components and sparsity parameter, using clr-transformed values that were centered and scaled. The coefficient values of the selected predictor features were then further subjected to a bootstrapping procedure and only predictors with non-zero coefficient values at 95% confidence intervals were considered as the final set of predictor features significantly correlated with the host dependent variable of interest **(S3 Fig and S8 Fig)**.

### RNA isolation from PAXgene tubes for RNA-seq

RNA isolation from the blood PAXgene tubes was done using the PAXgene nucleic acid purification kit (PreAnalytiX) according to manufacturer’s protocol. RNA-seq library preparation was done at the NYU School of Medicine Genome Technology Core using an automated stranded polyA enrichment library preparation protocol. Libraries were sequenced on a HiSeq 2000 (Illumina) with 2 × 50 cycles and for an average of 50 million reads per sample.

### RNA-seq reads processing

Raw RNA-seq reads were aligned to the reference human genome Grch37 and the Ensembl reference transcriptome Homo_sapiens.GRCh37.82.gtf with Tophat (version 2.1.0), with all parameters kept at default settings [68]. Reads with a mapping quality score (MAPQ) of less than 30 and reads from mitochondrial DNA were removed. The number of filtered reads was subsequently counted for each gene by htseq-count, with the parameters—mode = union and—stranded = reverse [69]. The resulting count matrix was used for downstream analyses (**S4 Fig**).

### Management of batch effect in RNA-seq data

The polyA-enriched RNA libraries were sequenced in five different batches. To manage potential batch effects, we first removed differentially expressed genes across the different pools at FDR 10%, defined using the likelihood ratio test (LRT) implemented in the R/Bioconductor package *DESeq2* [70]. We performed PCA on the 24819 genes that were retained after this procedure and observed that no particular batch of samples stood out **(S12A Fig)**. We then used these retained genes for downstream PCA, vector fitting and SPLS-regression procedures. However, since the *DESeq2* differential analysis procedure relies on the estimation of a moderated dispersion value based on a negative binomial model and that it was imperative to retain as many genes as possible for statistical power of the fitting procedure, we used the unfiltered count matrix (38176 non-singleton genes) for the differential comparisons between (1) Orang Asli (pre-deworming) vs. urban participants and (2) Pre-deworming vs. Post-deworming Orang Asli. We verified that the genes significantly differential post-deworming were not affected by batch effect **(S12B Fig)**. While a subset of genes differential from the Orang Asli vs. urban participants comparison appeared to have batch specific expression patterns **(S12C Fig)**, we have refrained from making conclusions specific to this subset of genes.

### RNA-seq data analyses

To perform PCA on the RNA-seq data, we further filtered the above count matrix (where genes differential by sequencing batch were removed) and kept only genes with more than 10 counts in at least 10% samples. We next performed regularized logarithmic (rlog) transformation on the filtered count data matrix generated as described above and removed genes with low variance using the *varFilter* function in the R/Bionconductor *genefilter* package [71]. These filtering steps resulted in 6076 genes, on which we performed PCA using the rlog-transformed count values.

To identify biological variables significantly associated with variations in blood transcriptional profiles, we used the same workflow as described above for 16S microbial variations, but replaced the PCoA scores on Bray-Curtis dissimilarity matrix with PCA scores on the rlog-transformed count values. The 93 biological variables were then fit to the PCA scores of the first two principal components using the *envfit* function in *vegan*. SPLS-regression of rlog-transformed RNA-seq count values on pre-deworming egg count was performed as described above for the SPLS-regression on 16S profiles, with the addition of a variance filtering step, where genes with coefficient of variance (CV) less than the median per-gene CV value were removed. Rlog-transformed count values were centered but not scaled for SPLS-regression model fitting.

Supervised differential analyses based on the negative binomial distribution with moderated dispersion value estimation were done with the workflow directly implemented through the *DESeq* function in the *DESeq2* package. We fit two separate models and performed two separate comparisons to identify differential genes between (1) Orang Asli participants (pre-deworming) vs. urban participants, as well as (2) post-deworming vs. pre-deworming among the Orang Asli participants. Significantly differential genes were defined using a false discovery rate (FDR) of 10% and |log_2_ fold change| greater than zero. Pathway enrichment analyses were done using QIAGEN’s Ingenuity Pathway Analysis software (IPA, www.Ingenuity.com) for genes with differential expression between Orang Asli (pre-deworming) and urban participants, using the “Human” option for the “Organism” parameter and keeping all other parameters at default settings.

### Quantitating the variability in blood immune response genes

We wanted to determine if the differences in immune response among the Orang Asli participants were greater than that among the urban participants (**S7 Fig**). We first identified all genes annotated as “Cytokine” (Gene Ontology identifier:0005125) or “Chemokine” (Gene Ontology identifier:0008009) based on the Gene Ontology database and defined these as immune response genes. Only immune response genes with at least 10 counts were included in the analysis, resulting in 82 immune response genes. We then extracted the rlog-transformed count values of these 82 immune response genes for quantitation of the variability in their expression levels. To compare the variation across the 82 immune response genes between the Orang Asli and the urban population, we first applied PCA to crease uncorrelated principal component variables. Using the first 20 principal components (~90% of the total variation), the first linear discriminant of the two populations were tested using the Fligner-Killeen test, where the null model states that both populations have equal variance. Next, we sought to identify specific immune response genes with differences in variance between the Orang Asli and urban participants, using the Fligner-Killeen test as well. We defined immune response genes with the largest differences in variance based on the Fligner-Killeen (median) χ^2^ test statistic and presented genes with a BH-adjusted p-value of <0.2.

### Linking the variations between the gut microbiota with blood immune genes

To relate variability in the immune response with specific members of the gut microbiota, we first summarized the 16S OTU count data at the “Order” taxonomic level. We then kept only orders with more than 10 counts in at least 10% samples and performed clr-transformation on the count data of the retained orders [37]. This resulted in 30 orders. We next performed PCA on the 82 immune response genes identified from above, using their unscaled rlog-transformed counts. To identify microbial orders significantly associated with blood immune response variations, we fit the clr-transformed count values of each order to the scores of the PCA (scores from PC1 and PC2) performed using expression levels of blood immune response genes, similar to the approach used to identify covariates associated with variations in gut microbiota and blood transcriptional profiles as described above.

### Identification of microbial OTUs and genes associated with changes in serum metal levels

To identify microbial OTUs and genes significantly associated with changes in serum metal levels pre- and post-deworming, we used the SPLS-regression workflow described above, but incorporated an additional step to account for the repeated measurements. We used a multivariate approach that took into account the mean effect of a perturbation (i.e. changes in serum metal levels) and the individual differential response to the same perturbation [72]. The “within-subject variation” can be extracted by subtracting off the between-subject variation from the mean perturbation effect, resulting in a data matrix where the values were directly proportional to the perturbation effect on each individual. We implemented this step directly using the *withinVariation* function from the R *mixOmics* package [73] and with a one-factor decomposition. This step was done on the clr-transformed OTU counts or the rlog-transformed gene counts, as well as the host covariate values (serum zinc and iron levels), pre- and post-deworming. SPLS-regression was then run on the within-subject values. Rlog-transformed gene counts were centered but not scaled for model fitting, but clr-transformed OTU counts were centered and scaled for model fitting.

### Immune cell deconvolution from blood transcriptional profiles

To ascertain immune cell type proportions from whole blood transcriptional profiles we applied the CIBERSORT [74] deconvolution algorithm to count tables normalized by sequencing depth from the blood transcriptional profile. The default LM22 matrix curated in the original CIBERSORT publication enabled identification of 22 immune cell types. To determine whether urban control, and Orang Asli pre-deworming and Orang Asli post-deworming populations had significantly different sample means, we performed a bootstrapped (999 sample) one-way ANOVA. Statistical significance was defined as a p-value <0.05. Tukey’s test with multiple comparisons correction was done when the ANOVA result was statistically significant. Methodology is partially based on [74].

### Zinc stimulation of monocyte-derived macrophages

Monocyte-derived macrophages were prepared *ex vivo* from human PBMCs cultured in MDDC medium (RPMI + 10% FBS +10mM Hepes +1:100P/S) at 1x10^6^ cells/mL. GMCSF was added at a 1x concentration, and cells were incubated in 20mL plates at 37 degrees Celsius and 5% CO_2_ for four days. An additional 1x CO_2_ was added on the second day. On the 4^th^ day, cells were collected from culture plates, and diluted to 1.25x10^6^ cells/mL. We added 80ul of cells to wells in a 96-well flat-bottom plate, along with cytokines diluted in RPMI + 10% FBS. Recombinant human IL-4 and IL-13 were from R&D Systems, and Zinc sulfate was from Sigma-Aldrich. IL-4 was used at a concentration of 1000 IU/ml (34.5 ng/ml), as well as 1:10 and 1:100 dilutions. IL-13 was used at a concentration of 100 IU/ml (128.2 ng/ml), as well as 1:10 and 1:100 dilutions. Zinc sulfate was diluted to a concentration of 100uM (0.03ng/ml), and found to be uncontaminated with LPS relative to the media in which it was diluted. Cells were incubated for 4 hours, then concentrated and collected in RLT buffer (Qiagen) and stored at -80 degrees Celsius until RNA isolation. Total RNA was isolated using RNeasy Plus Mini Kit (Qiagen) with in-column DNase treatment (Qiagen). The CEL-Seq single-cell RNAseq analysis method (Hashimshony et al. 2016) was performed for three lanes of a paired-end 50 Illumina HiSeq 4000 run. Per-read per-lane FASTQ files were generated using the bcl2fastq2 Conversion software (v.2.2.0) to convert per-cycle BCL base call output files produced by the sequencing instrument into the FASTQ format, and the FASTQ files were then further split based on corresponding CEL-seq barcodes for each sample using the CEL-Seq demultiplexer, “bc_demultiplex”. The CEL-Seq wrapper for the alignment program, Bowtie2 (v.2.3.4.1), was used for mapping reads of 264 demultiplexed human CEL-Seq samples to the human reference genome hg19; and the CEL-Seq wrapper for the HTSeq-count script of the Python (v.2.7.15) package, HTSeq, was used to generate the matrix of read counts for annotated genomic features. This resulted in a final median read depth of 1,707,022 mapped reads per sample, covering a median of 10,210 genes per sample. PCA was performed using 1835 genes with high variance, defined using the varFilter function in the genefilter package with default parameters, which keeps only features with variance inter-quartile range > 0.85. Gene Ontology annotation terms associated with each of the genes were fetched through the R package GO.db [75].

## Supporting information

S1 Fig PCoA comparing Orang Asli and urban control participants based on Bray-Curtis distances from 16s rRNA gene sequencing. (TIF)

S2 Fig Methodology for identification of correlations between nutritional and blood variables with OTU composition. (TIF)

S3 Fig Schematic for SPLS-regression for cross-sectional analyses. (TIF)

S4 Fig RNA-seq analytical methodology for identification of differences in transcriptional profiles between Orang Asli and urban participants. (TIF)

S5 Fig Linear regression of changes in cell population proportions and the difference between the natural log of *Trichuris* egg count post- and pre-deworming. (TIF)

S6 Fig Identification of correlated cytokine and chemokines with OTU composition. (TIF)

S7 Fig Distribution of first linear discriminant scores derived from the first 20 principal components of 82 immune genes (~90% total variation) from 42 Orang Asli participants and 18 urban participants. Only samples with matching RNA-seq and 16S microbiota profiles were included in this analysis. P-value (0.11) was based on the Fligner-Killeen test.
(TIF)

S8 Fig Schematic for SPLS-regression for repeated measurements analyses. (TIF)

S9 Fig Genes contributing most strongly to PC1 in PCA plot of samples stimulated with and without zinc. A) Percent contributions to PC1 be the top 50 genes. B) Heatmap of gene expression of these top 50 genes contributing to PC1. Nine are downregulated in the presence of zinc and the remainder are upregulated. Results are fairly consistent across the 7 samples unstimulated with zinc, versus the 7 samples stimulated with zinc. Many genes are metallothioneins.
(TIF)

S10 Fig Distribution of baseline Trichuris egg burden according to age and gender. Note that age (but not gender) appears to be associated with *Trichuris* burden (children tend to have a higher load).
(TIF)

S11 Fig PCoA comparing OA and urban control participants based on Bray-Curtis distances from 16s rRNA gene sequencing, with samples additionally coded based on protozoan infections. (TIF)

S12 Fig Management of batch effect in RNA-seq data. A) PCA based on 24819 genes to look for batch effects. B) Clustered heatmap to determine whether differentially expressed genes (comparing Orang Asli participants pre-deworming and post-deworming) were affected by pooling/run batch effects. C) Clustered heatmap to determine whether differentially expressed genes (comparing Orang Asli participants pre-deworming and urban participants) were affected by pooling/run batch effects.
(TIF)

S1 Table List of blood variables measured from participants. (DOCX)

S2 Table Sample sizes for all analyses. (DOCX)

S3 Table Summary of demographic and infection-status characteristics of the sample. (DOCX)

S4 Table Twenty-nine genes that were significantly altered by deworming, were directionally more similar post-deworming to urban participants, and were associated with immune system processes based on gene ontology. (DOCX)

S5 Table Comparisons between CIBERSORT cell-type proportions between study populations for all 22 CIBERSORT cell populations. * Values are reported as mean proportion +- standard error. ** Not significant
(DOCX)

S6 Table Blood and dietary variables and their correlation with pre-deworming *Trichuris* egg counts. (DOCX)
