# Supplementary figures and images for: Linking the effects of helminth infection, diet and the gut microbiota with human whole-blood signatures
Source: PLoS Pathog. 2019 Dec 16;15(12):e1008066. doi: 10.1371/journal.ppat.1008066 (PMC6913942; doi:10.1371/journal.ppat.1008066)

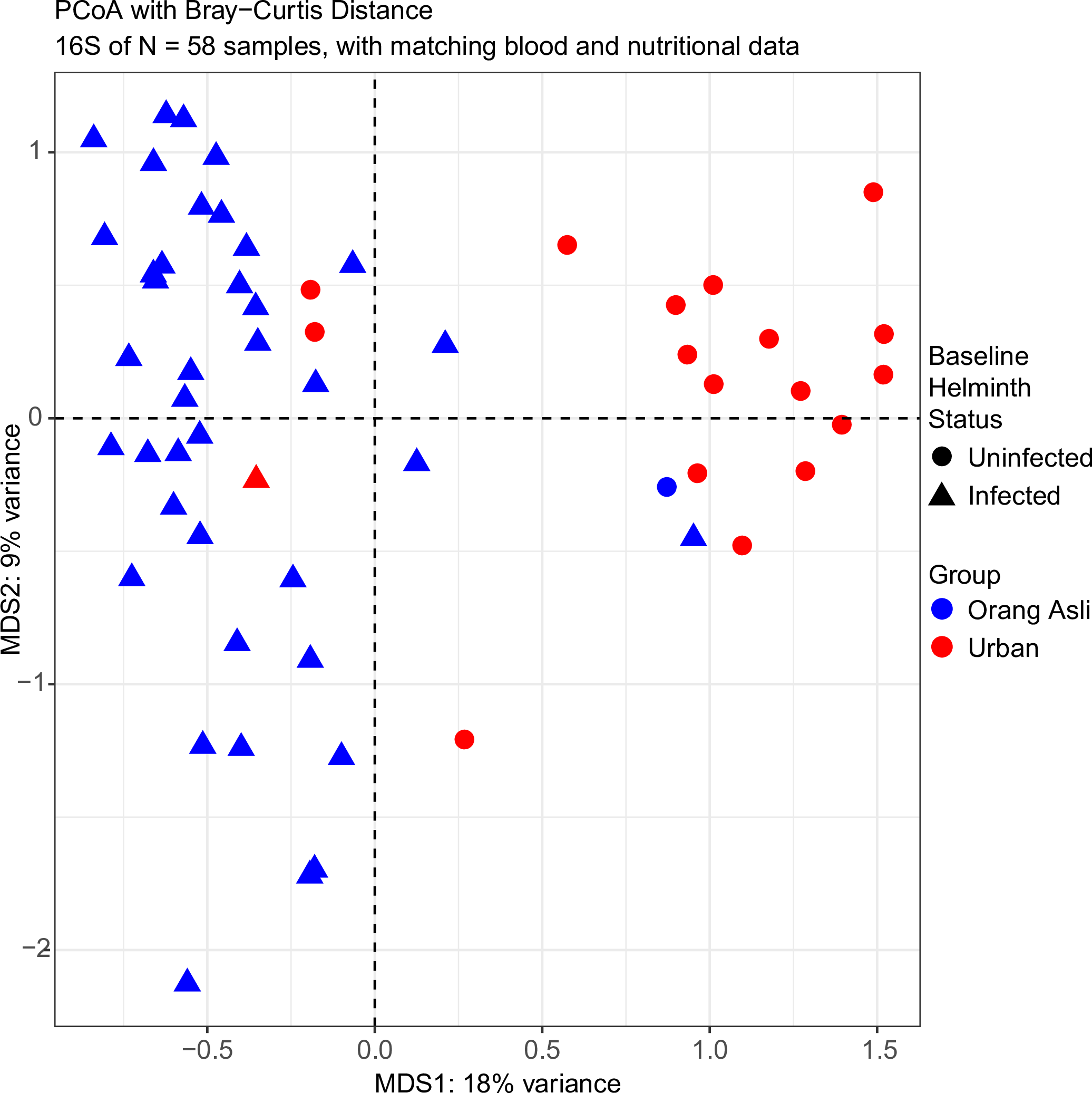

Supplement: S1 Fig — (TIF) [file ppat.1008066.s001.tif]

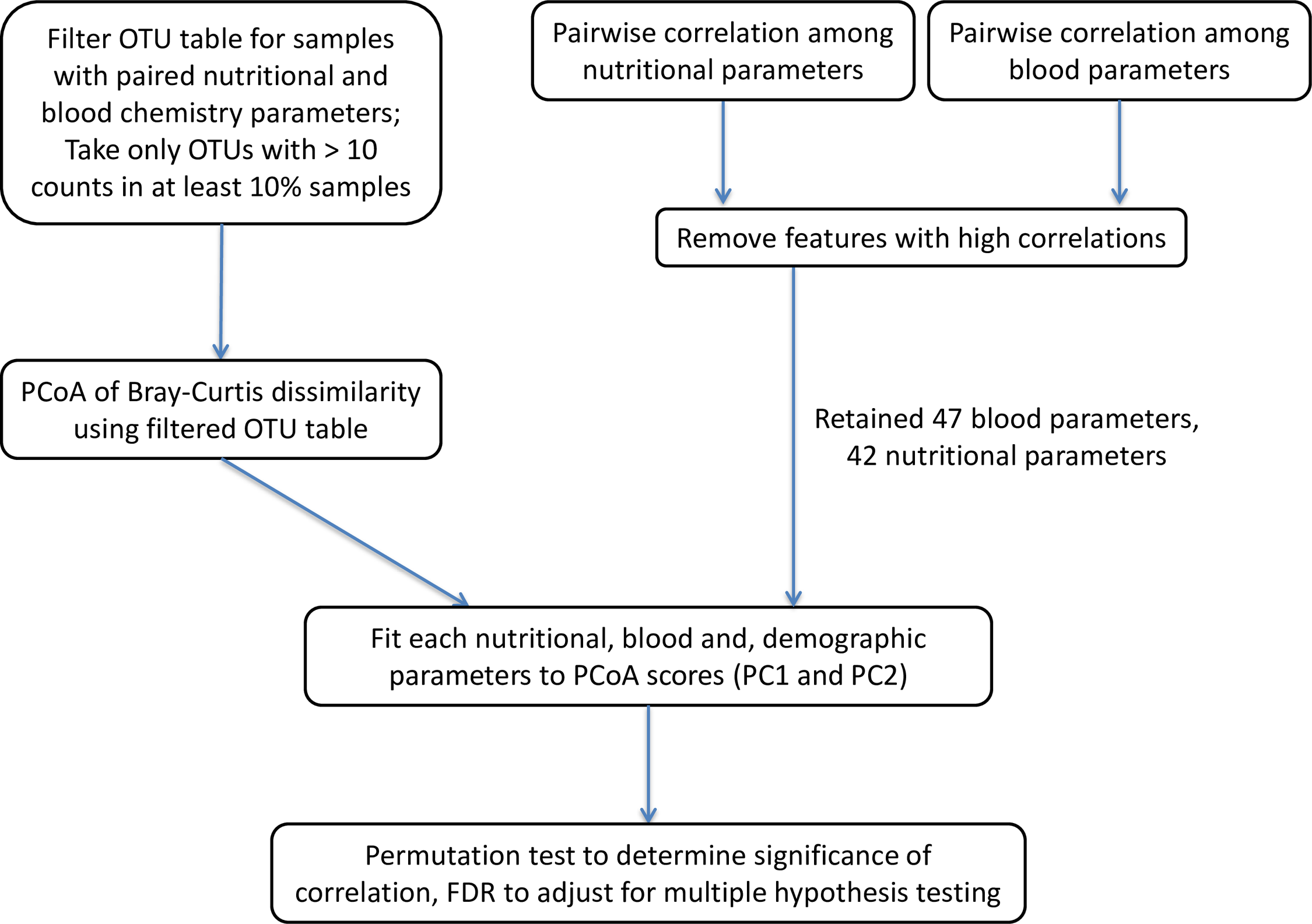

Supplement: S2 Fig — (TIF) [file ppat.1008066.s002.tif]

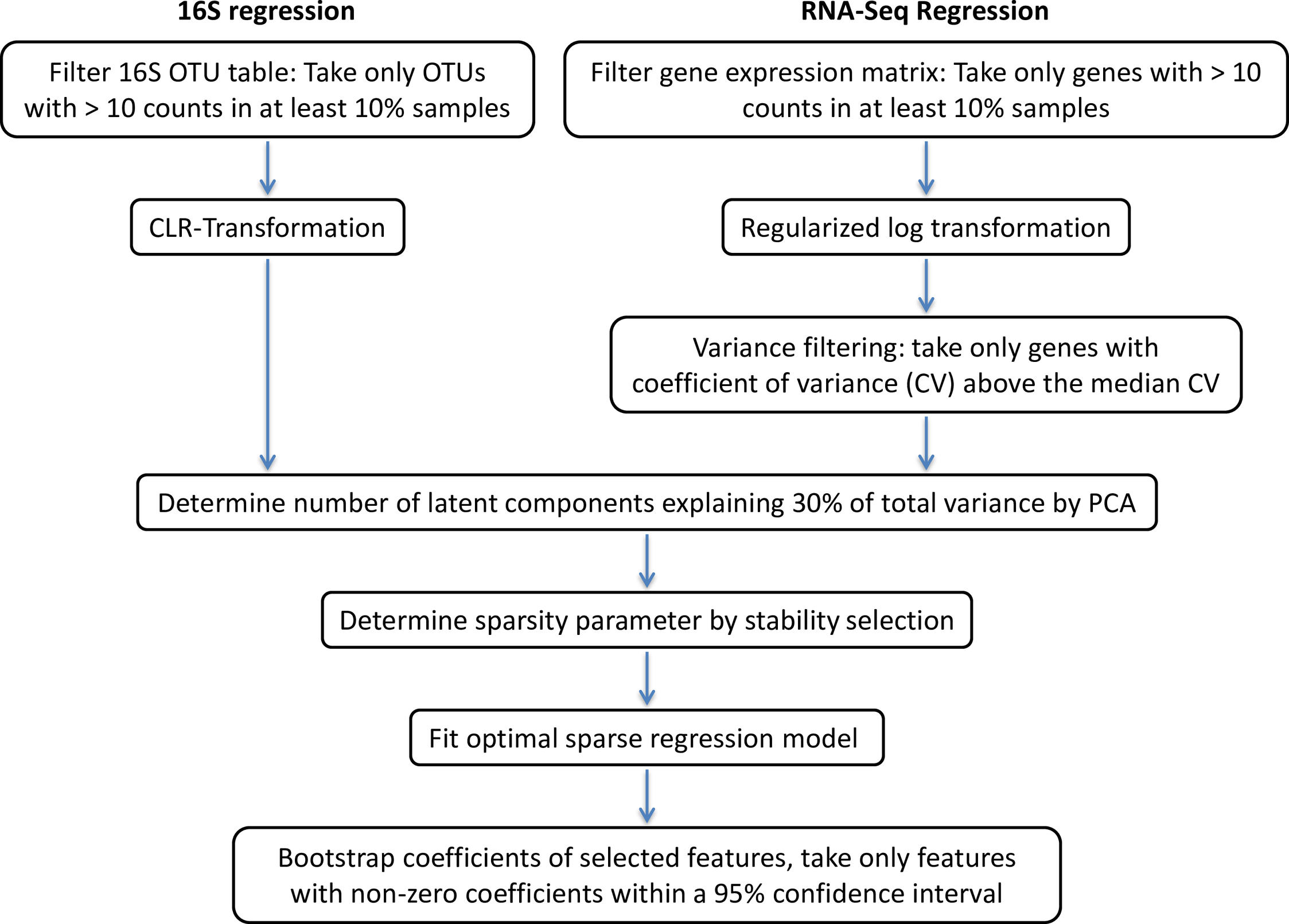

Supplement: S3 Fig — (TIF) [file ppat.1008066.s003.tif]

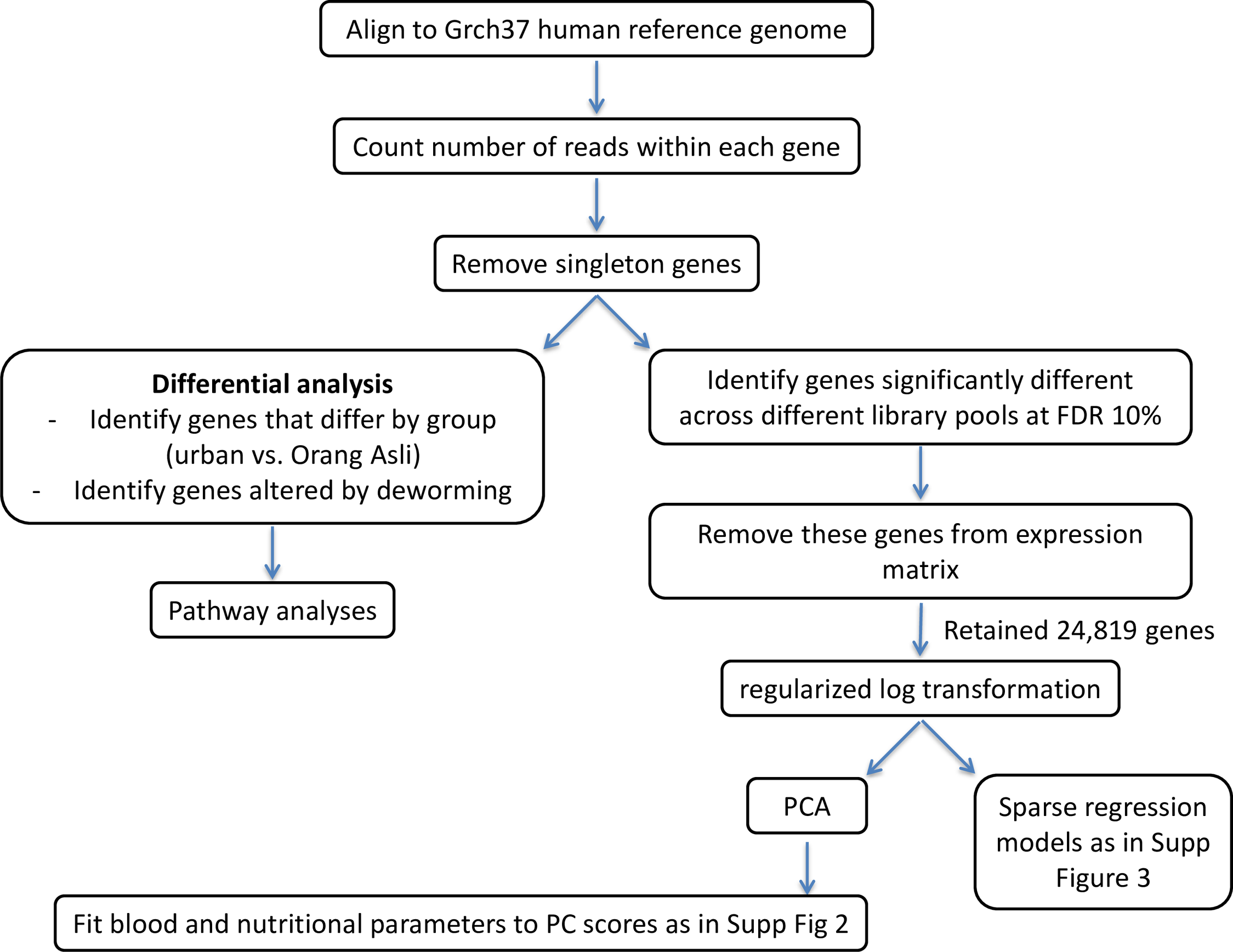

Supplement: S4 Fig — (TIF) [file ppat.1008066.s004.tif]

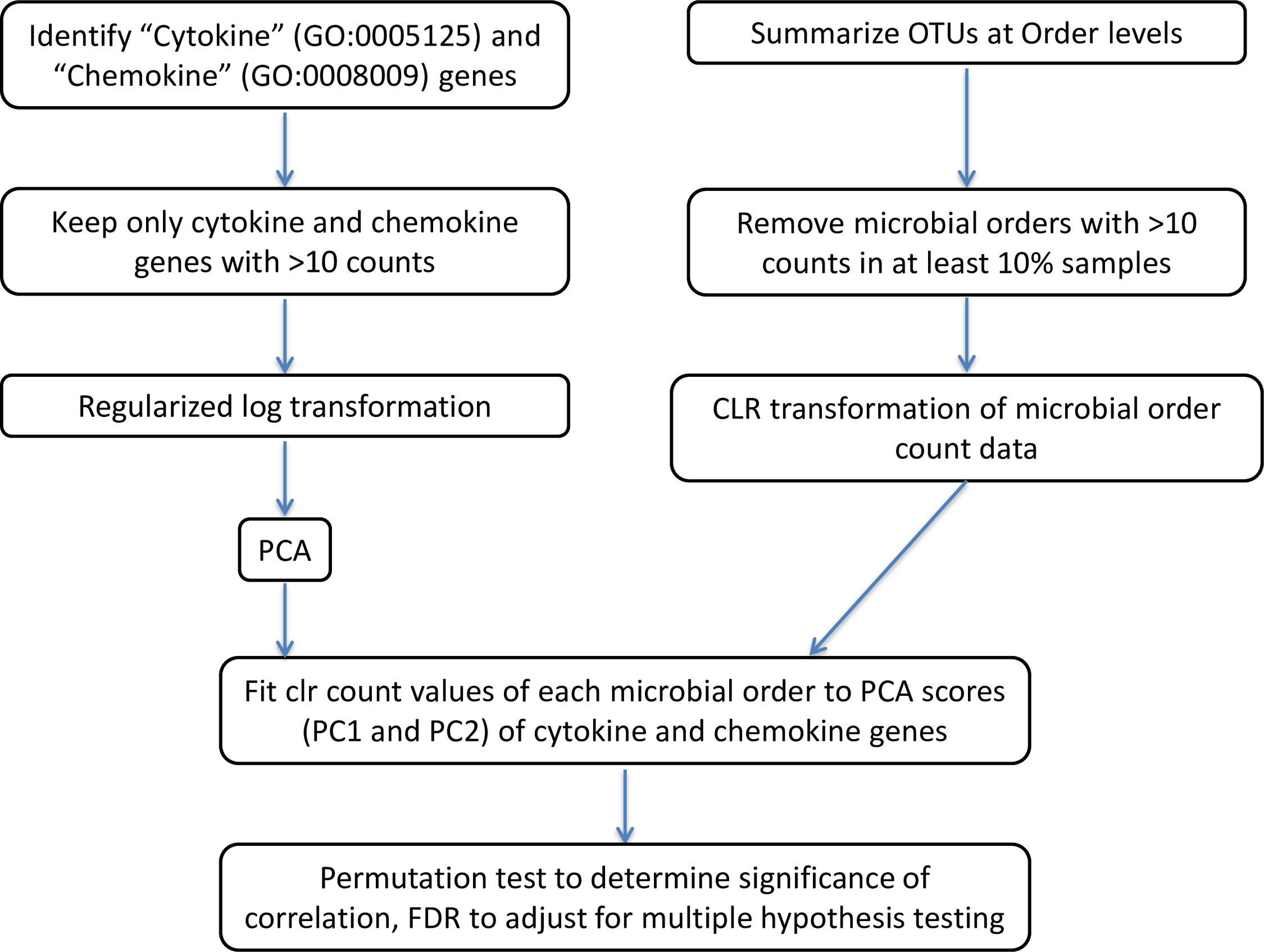

Supplement: S5 Fig — (TIF) [file ppat.1008066.s005.tif]

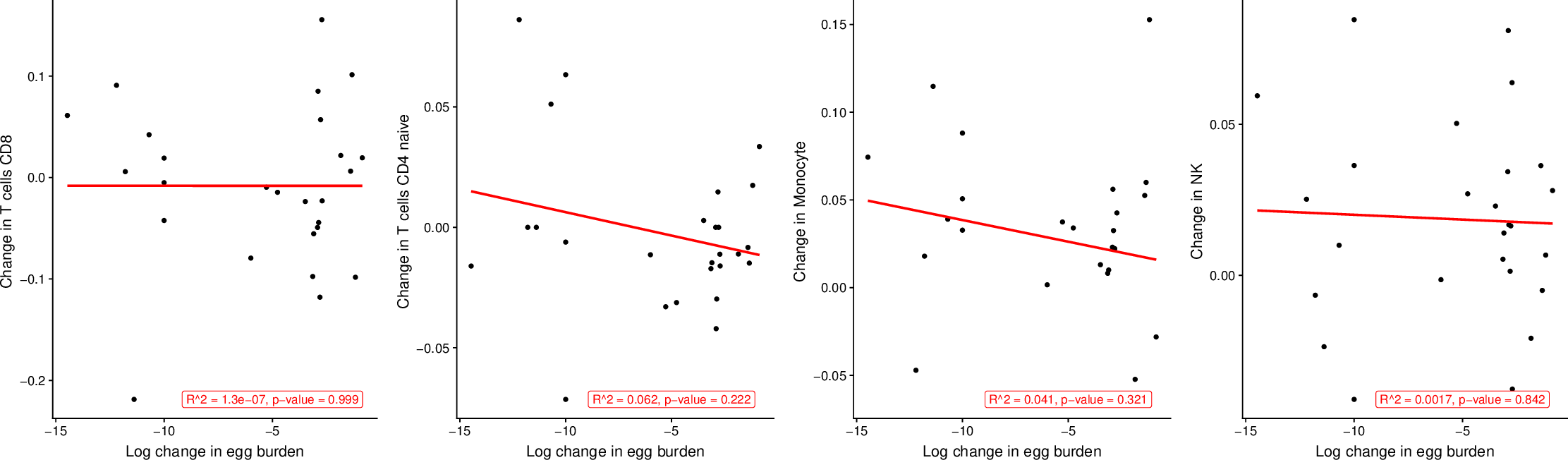

Supplement: S6 Fig — (TIF) [file ppat.1008066.s006.tif]

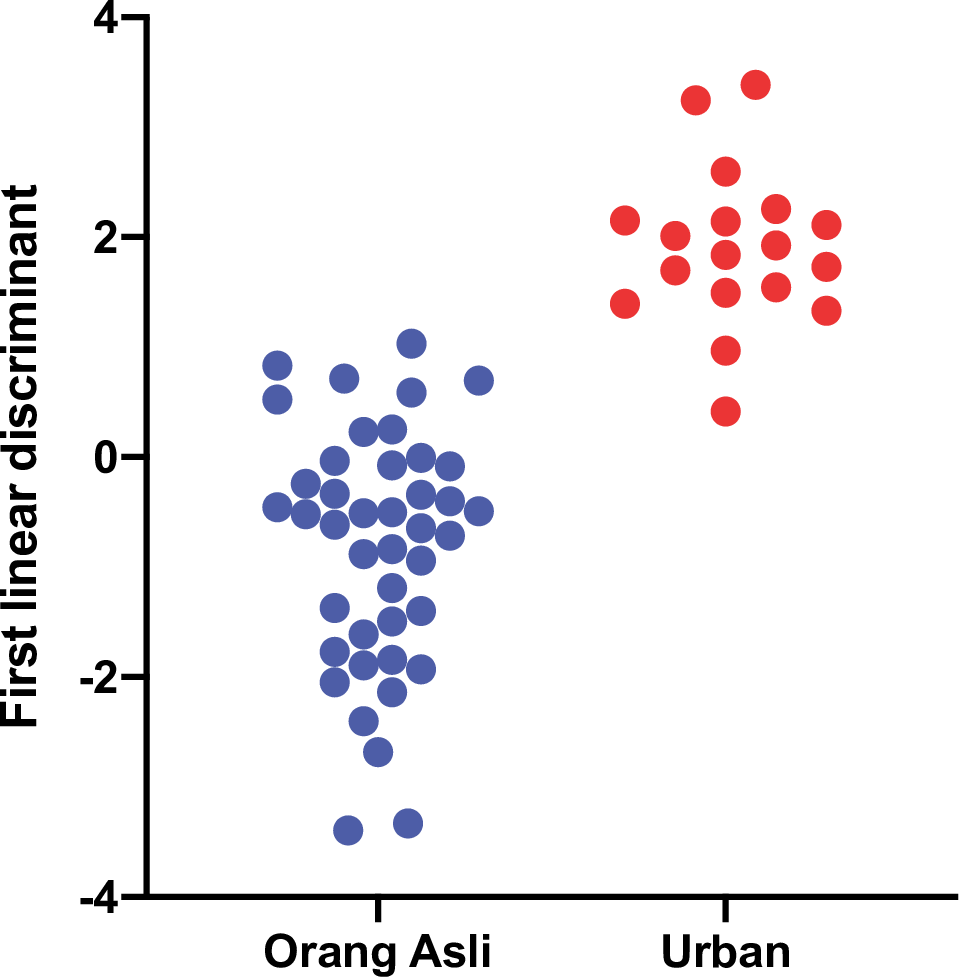

Supplement: S7 Fig — Only samples with matching RNA-seq and 16S microbiota profiles were included in this analysis. P-value (0.11) was based on the Fligner-Killeen test. (TIF) [file ppat.1008066.s007.tif]

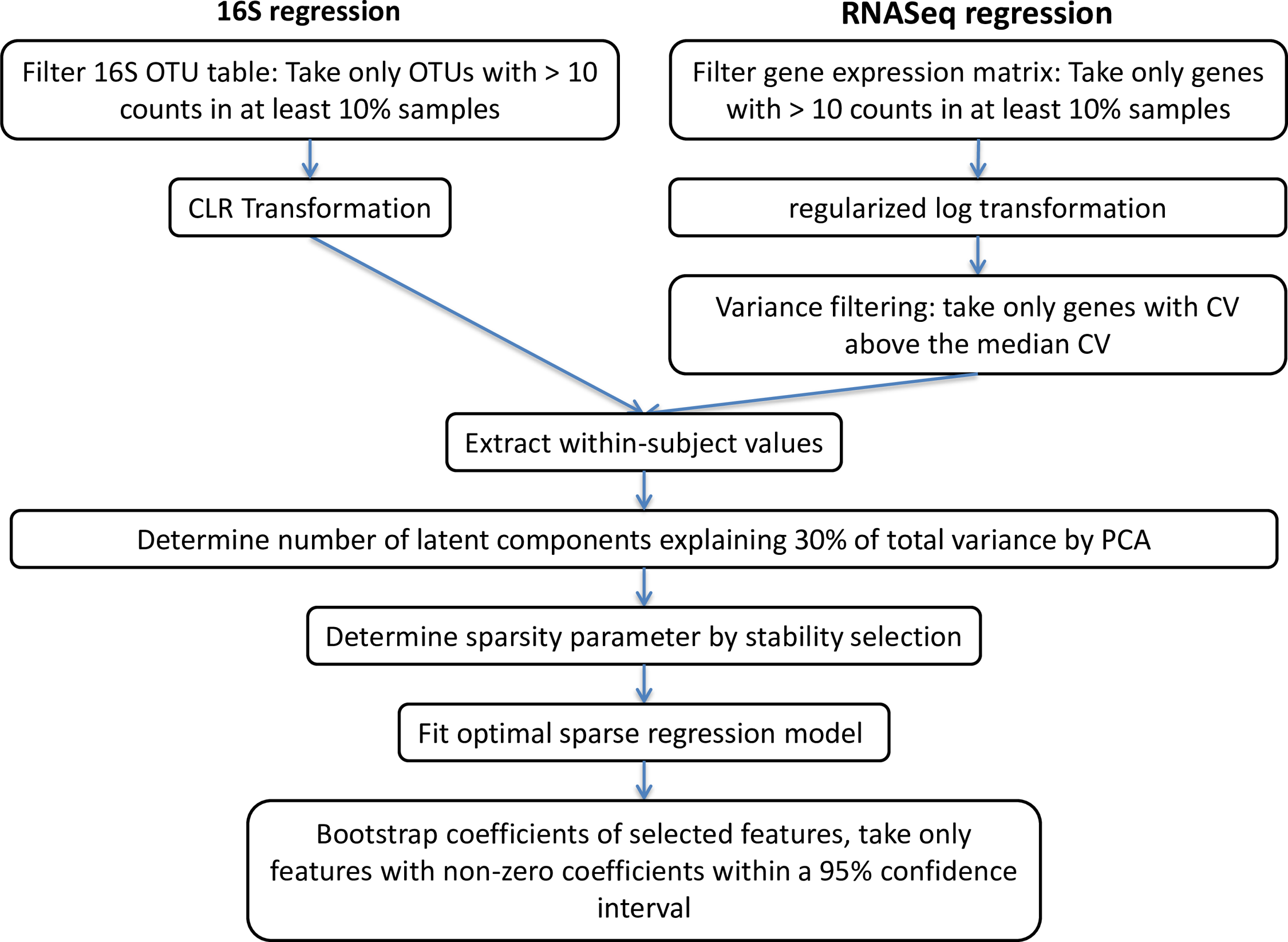

Supplement: S8 Fig — (TIF) [file ppat.1008066.s008.tif]

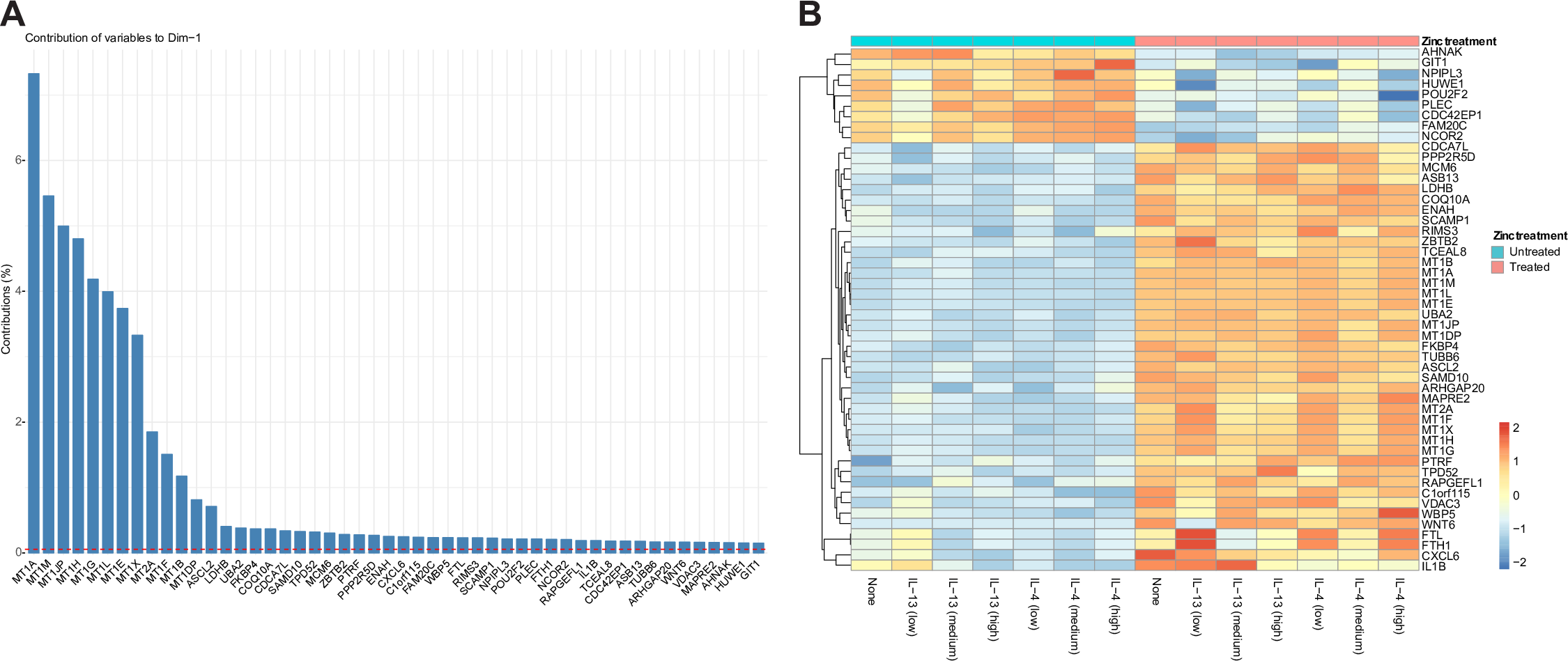

Supplement: S9 Fig — A) Percent contributions to PC1 be the top 50 genes. B) Heatmap of gene expression of these top 50 genes contributing to PC1. Nine are downregulated in the presence of zinc and the remainder are upregulated. Results are fairly consistent across the 7 samples unstimulated with zinc, versus the 7 samples stimulated with zinc. Many genes are metallothioneins. (TIF) [file ppat.1008066.s009.tif]

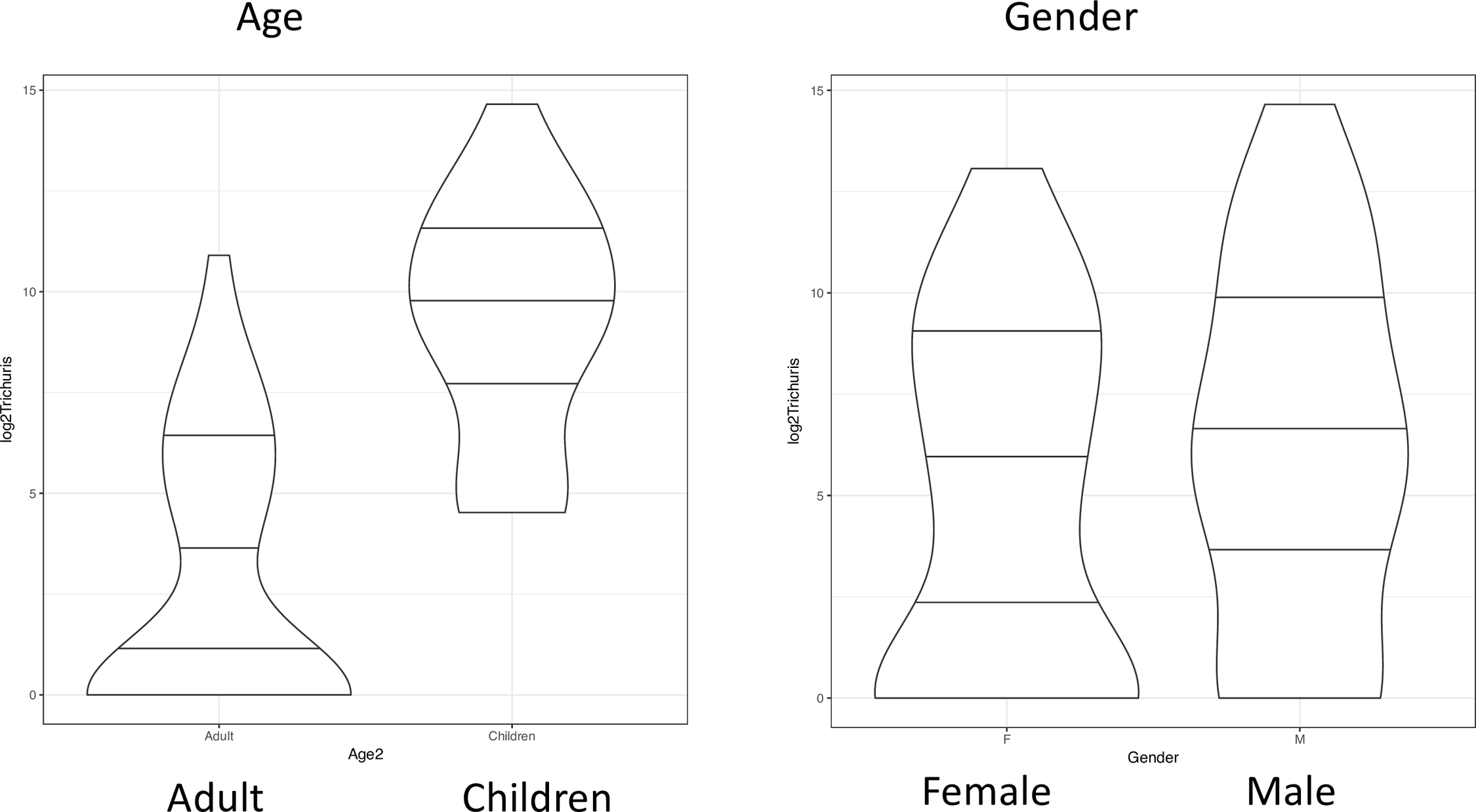

Supplement: S10 Fig — Note that age (but not gender) appears to be associated with Trichuris burden (children tend to have a higher load). (TIF) [file ppat.1008066.s010.tif]

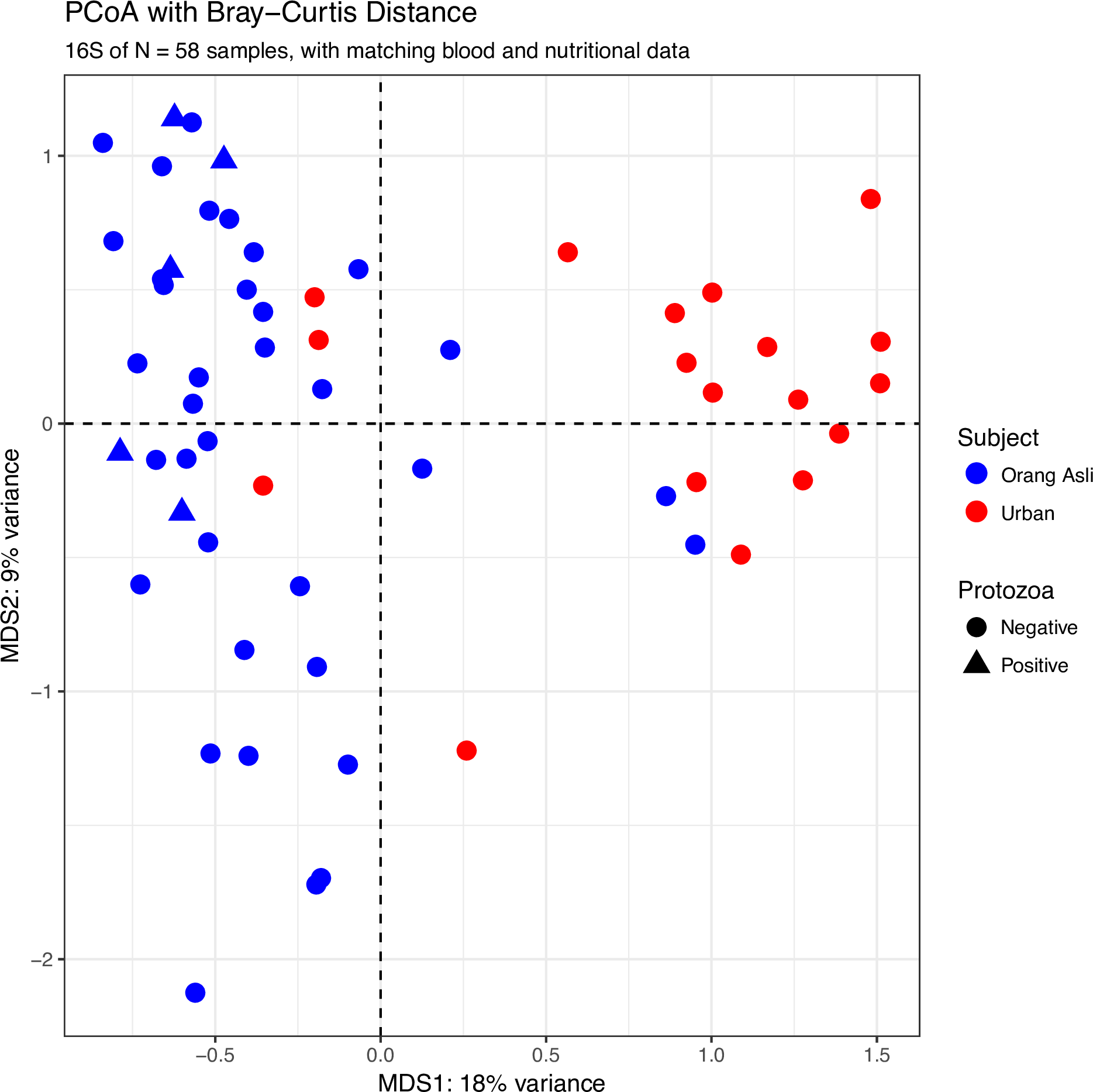

Supplement: S11 Fig — (TIF) [file ppat.1008066.s011.tif]

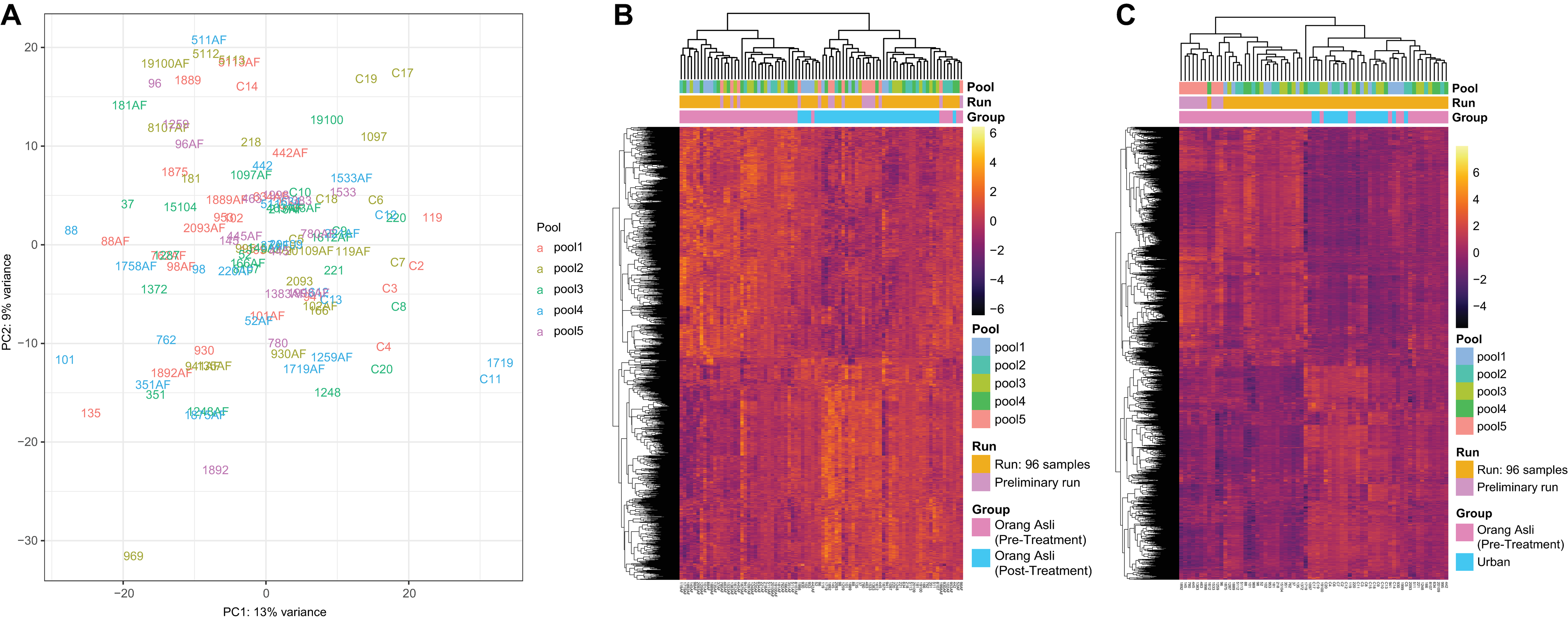

Supplement: S12 Fig — A) PCA based on 24819 genes to look for batch effects. B) Clustered heatmap to determine whether differentially expressed genes (comparing Orang Asli participants pre-deworming and post-deworming) were affected by pooling/run batch effects. C) Clustered heatmap to determine whether differentially expressed genes (comparing Orang Asli participants pre-deworming and urban participants) were affected by pooling/run batch effects. (TIF) [file ppat.1008066.s012.tif]
