# Supplementary material for: Linking the effects of helminth infection, diet and the gut microbiota with human whole-blood signatures
Source: PLoS Pathog. 2019 Dec 16;15(12):e1008066. doi: 10.1371/journal.ppat.1008066 (PMC6913942; doi:10.1371/journal.ppat.1008066)
Supplement: S1 Table — (DOCX) [file ppat.1008066.s013.docx]

**Table S1. List of blood variables measured from participants**

| T_RBC |
| --- |
| Hb |
| PCV |
| MCV |
| MCH |
| MCHC |
| RDW |
| T_WBC |
| Polymorphs |
| Lymphocytes |
| Monocytes |
| Eosinophils |
| Basophils |
| ESR |
| Platelet |
| Sodium |
| Potassium |
| Chloride |
| Urea |
| Creatinine |
| Uric_Acid |
| Glucose |
| Calsium |
| Phosphorus |
| T_Protein |
| Albumin |
| Globulin |
| A_G_Ratio |
| T_Bilirubin |
| SGOT_AST |
| SGPT_ALT |
| GGT |
| Alkaline_Phosphatase |
| T_Cholesterol |
| Triglycerides |
| HDL |
| LDL |
| T_Chol_HDL_Ratio |
| Serum_TIBC |
| Serum_Iron |
| Serum_Ferritin |
| Iron_Saturation |
| Cu |
| Zn |
