# Supplementary material for: Linking the effects of helminth infection, diet and the gut microbiota with human whole-blood signatures
Source: PLoS Pathog. 2019 Dec 16;15(12):e1008066. doi: 10.1371/journal.ppat.1008066 (PMC6913942; doi:10.1371/journal.ppat.1008066)
Supplement: S2 Table — (DOCX) [file ppat.1008066.s014.docx]

**Table S2. Sample sizes for all analyses.**

| **Figure** | **Number of samples** |
| --- | --- |
| 2C | 49 pre-deworming OA, 18 urban subjects with dietary profiles |
| 2D | 46 pre-deworming OA, 18 urban subjects with blood chemistry profiles |
| 3A, 3F | 40 pre-deworming OA, 18 urban subjects with matching 16S, blood chemistry and dietary profiles |
| 3B | 46 pre-deworming OA, 18 urban subjects with blood chemistry profiles |
| 3C | 49 pre-deworming OA, 18 urban subjects with dietary profiles |
| 3D | 42 pre-deworming OA with matching 16S and dietary fiber level |
| 3E | 42 pre-deworming OA with matching 16S and pre-deworming *Trichuris* egg count |
| 4A,4B | 46 pre-deworming OA, 18 urban subjects with matching blood RNA-Seq, blood chemistry profiles and dietary profiles |
| 4C | 49 pre-deworming OA with matching blood RNA-Seq and pre-deworming *Trichuris* egg count |
| 5A-5C | 49 pre-deworming OA, 18 urban subjects with blood RNA-Seq |
| 5D | 42 pairs of pre- and post-deworming OA with paired blood RNA-Seq (42 x 2 = 84 blood RNA-Seq samples) |
| 5E,5F | 18 urban subjects, 49 pre-deworming OA and 44 post-deworming OA with blood RNA-Seq |
| 7A-7D | 42 pre-deworming OA with matching 16S and blood RNA-Seq profiles |
| 8A: 3 group comparison: Zinc | 18 urban subjects, 46 pre-deworming OA, 33 post-deworming OA with blood zinc levels |
| 8A: 3 group comparison: Iron | 18 urban subjects, 46 pre-deworming OA, 34 post-deworming OA with blood iron levels |
| 8A: 3 group comparison: Globulin | 18 urban subjects, 46 pre-deworming OA, 34 post-deworming OA with blood globulin levels |
| 8A: Paired comparison: Zinc | 30 pairs of pre- and post-deworming OA with paired blood zinc levels |
| 8A: Paired comparison: Iron | 31 pairs of pre- and post-deworming OA with paired blood iron levels |
| 8A: Paired comparison: Globulin | 31 pairs of pre- and post-deworming OA with paired blood globulin levels |
| 8B | 27 pairs of pre- and post-deworming OA with paired and matching 16S profiles and blood zinc levels (27 x 2 = 54 16S profiles with matching blood zinc levels) |
| 8C | 28 pairs of pre- and post-deworming OA with paired and matching 16S profiles and blood iron levels (28 x 2 = 56 16S profiles with matching blood iron levels) |
| 8D | 30 pairs of pre- and post-deworming OA with paired and matching blood RNA-Seq and blood zinc levels (30 x 2 = 60 blood RNA-Seq profiles with matching blood zinc levels) |
| 8E | 31 pairs of pre- and post-deworming OA with paired and matching blood RNA-Seq and blood iron levels (31 x 2 = 62 blood RNA-Seq profiles with matching blood iron levels) |
