# Supplementary material for: Linking the effects of helminth infection, diet and the gut microbiota with human whole-blood signatures
Source: PLoS Pathog. 2019 Dec 16;15(12):e1008066. doi: 10.1371/journal.ppat.1008066 (PMC6913942; doi:10.1371/journal.ppat.1008066)
Supplement: S3 Table — (DOCX) [file ppat.1008066.s015.docx]

**Table S3. Summary of demographic and infection-status characteristics of the sample**

|  | **Urban** | **Orang Asli** |
| --- | --- | --- |
| Total | 18 | 49 |
| Male | 5 | 24 |
| Female | 13 | 25 |
| Median age (years) | 24 | 15 |
| *Trichuris*-infected | 0 | 44 |
| *Ascaris*-infected | 0 | 4 |
| Hookworm-infected | 1 | 19 |
| *Entamoeba*-infected | 0 | 4 |
| *Giardia*-infected | 0 | 1 |
| Infected with *Trichuris* and hookworm | 0 | 17 |
