# Supplementary material for: Linking the effects of helminth infection, diet and the gut microbiota with human whole-blood signatures
Source: PLoS Pathog. 2019 Dec 16;15(12):e1008066. doi: 10.1371/journal.ppat.1008066 (PMC6913942; doi:10.1371/journal.ppat.1008066)
Supplement: S4 Table — (DOCX) [file ppat.1008066.s016.docx]

**Table S4. Twenty-nine genes that were significantly altered by deworming, were directionally more similar post-deworming to urban participants, and were associated with immune system processes based on gene ontology.**

| Gene | Ensemble ID | log2 Fold Change post- vs pre-deworming | padj | log2 Fold Change baseline Orang Asli vs Urban | padj |
| --- | --- | --- | --- | --- | --- |
| ATP8B4 | ENSG00000104043 | -0.4190692 | 0.03117855 | 0.68660791 | 0.00330244 |
| CCR2 | ENSG00000121807 | -0.5117247 | 0.00059835 | 0.40458511 | 0.05565261 |
| CD151 | ENSG00000177697 | -0.3718546 | 0.00530715 | 0.40552487 | 0.07579716 |
| CD36 | ENSG00000135218 | -0.4656522 | 0.00466326 | 0.63597972 | 0.00682746 |
| CLEC12A | ENSG00000172322 | -0.3882786 | 0.08926738 | 0.74967884 | 0.056172 |
| CTSC | ENSG00000109861 | -0.2376843 | 0.04800793 | 0.29811533 | 0.02007797 |
| DOK2 | ENSG00000147443 | -0.2693525 | 0.02605178 | 0.33801789 | 0.03691659 |
| DROSHA | ENSG00000113360 | 0.35948352 | 0.07757983 | -0.6057345 | 0.00022952 |
| ERAP2 | ENSG00000164308 | -0.3105907 | 0.0460977 | 0.60470751 | 0.03651912 |
| EXOSC9 | ENSG00000123737 | 0.33202374 | 0.0240047 | -0.4222843 | 0.00835693 |
| HLA-DRA | ENSG00000204287 | -0.3292215 | 0.01315949 | 0.33988259 | 0.04270054 |
| HSD3B7 | ENSG00000099377 | -0.6081014 | 0.00421087 | 0.7694735 | 0.01061046 |
| IDO1 | ENSG00000131203 | -0.6576586 | 0.07663575 | 1.31286513 | 0.00493151 |
| IGF1R | ENSG00000140443 | 0.35861509 | 0.06135699 | -0.5930529 | 0.00887124 |
| IGHE | ENSG00000211891 | -0.7118847 | 0.03087115 | 2.404165 | 3.42E-06 |
| ITFG2 | ENSG00000111203 | 0.25749032 | 0.05249893 | -0.2659283 | 0.07303185 |
| LY86 | ENSG00000112799 | -0.2740113 | 0.05797646 | 0.33204033 | 0.06608695 |
| LYZ | ENSG00000090382 | -0.2947606 | 0.0657846 | 0.60021218 | 0.00983129 |
| MGST1 | ENSG00000008394 | -0.3494213 | 0.0752468 | 0.4112344 | 0.05291536 |
| MTOR | ENSG00000198793 | 0.20806001 | 0.09498251 | -0.3607213 | 0.0093839 |
| PIK3R6 | ENSG00000174083 | -0.4422888 | 0.03117855 | 0.57808693 | 0.05159481 |
| PLAC8 | ENSG00000145287 | -0.3143411 | 0.05241751 | 0.50918807 | 0.02432919 |
| POLR3C | ENSG00000186141 | 0.19185905 | 0.0657846 | -0.3736603 | 4.26E-05 |
| RRAS | ENSG00000126458 | -0.4592928 | 0.00929559 | 0.44462532 | 0.04898148 |
| SDC2 | ENSG00000169439 | 0.91074757 | 0.04487807 | -0.8827407 | 0.02209061 |
| SFPQ | ENSG00000116560 | 0.22106995 | 0.09350019 | -0.4150925 | 0.00145705 |
| STOM | ENSG00000148175 | -0.2860114 | 0.01235543 | 0.82999517 | 9.81E-08 |
| STON2 | ENSG00000140022 | -0.7548731 | 0.08985316 | 1.05358734 | 0.00872341 |
| TNRC6C | ENSG00000078687 | 0.28266056 | 0.05452567 | -0.316088 | 0.06992117 |
