# Supplementary material for: Linking the effects of helminth infection, diet and the gut microbiota with human whole-blood signatures
Source: PLoS Pathog. 2019 Dec 16;15(12):e1008066. doi: 10.1371/journal.ppat.1008066 (PMC6913942; doi:10.1371/journal.ppat.1008066)
Supplement: S5 Table — * Values are reported as mean proportion +- standard error. ** Not significant (DOCX) [file ppat.1008066.s017.docx]

**Table S5. Comparisons between CIBERSORT cell-type proportions between study populations for all 22 CIBERSORT cell populations.**

| Cell Type | Urban* | Pre-deworming | Post-deworming | ANOVA | Urban vs Pre | Urban vs Post | Pre vs Post |
| --- | --- | --- | --- | --- | --- | --- | --- |
| B cells naive | 0.0276 ± 0.0267 | 0.0355 ± 0.0269 | 0.0313 ± 0.0232 | 0.488 | NS** | NS | NS |
| B cells memory | 0.0105 ± 0.0169 | 0.0097 ± 0.0136 | 0.009 ± 0.0147 | 0.912 | NS | NS | NS |
| Plasma cells | 0 ± 0 | 0 ± 0 | 0 ± 0 | NA | NS | NS | NS |
| T cells CD8 | 0.1473 ± 0.0599 | 0.1909 ± 0.0664 | 0.1861 ± 0.0557 | 0.035 | p < 0.0267 | p < 0.0598 | p < 0.9232 |
| T cells CD4 naive | 0.0478 ± 0.0358 | 0.0223 ± 0.0277 | 0.0236 ± 0.0231 | 0.004 | p < 0.0051 | p < 0.0099 | p < 0.9722 |
| T cells CD4 memory resting | 0.0082 ± 0.0129 | 0.0234 ± 0.0338 | 0.0204 ± 0.0317 | 0.222 | NS | NS | NS |
| T cells CD4 memory activated | 5e-04 ± 0.0019 | 0.0022 ± 0.0057 | 0.004 ± 0.0107 | 0.225 | NS | NS | NS |
| T cells follicular helper | 0 ± 0 | 0 ± 0 | 1e-04 ± 8e-04 | 0.328 | NS | NS | NS |
| T cells regulatory | 0.0741 ± 0.024 | 0.0733 ± 0.0209 | 0.0662 ± 0.0214 | 0.254 | NS | NS | NS |
| T cells gamma delta | 0 ± 0 | 0 ± 0 | 0 ± 0 | NA | NS | NS | NS |
| NK cells resting | 0.0938 ± 0.0373 | 0.074 ± 0.0495 | 0.0809 ± 0.0457 | 0.322 | NS | NS | NS |
| NK cells activated | 0.0283 ± 0.0238 | 0.0463 ± 0.0244 | 0.0325 ± 0.0192 | 0.013 | p < 0.0236 | p < 0.8111 | p < 0.0211 |
| Monocytes | 0.207 ± 0.0438 | 0.194 ± 0.0483 | 0.1859 ± 0.0449 | 0.278 | NS | NS | NS |
| Macrophages M0 | 0.0131 ± 0.0133 | 0.0026 ± 0.0074 | 0.0016 ± 0.0059 | 0.001 | p < 1e-04 | p < 0 | p < 0.8321 |
| Macrophages M1 | 0 ± 0 | 0 ± 0 | 0 ± 0 | NA | NS | NS | NS |
| Macrophages M2 | 0 ± 0 | 0.0014 ± 0.0034 | 6e-04 ± 0.0027 | 0.214 | NS | NS | NS |
| Dendritic cells resting | 0 ± 0 | 0 ± 2e-04 | 3e-04 ± 0.001 | 0.05 | NS | NS | NS |
| Dendritic cells activated | 0.0018 ± 0.0035 | 8e-04 ± 0.0029 | 2e-04 ± 7e-04 | 0.042 | p < 0.2879 | p < 0.039 | p < 0.3835 |
| Mast cells resting | 0.0254 ± 0.0237 | 0.0164 ± 0.0091 | 0.02 ± 0.0137 | 0.079 | NS | NS | NS |
| Mast cells activated | 0 ± 0 | 2e-04 ± 0.0017 | 0 ± 0 | 0.607 | NS | NS | NS |
| Eosinophils | 0 ± 0 | 0.0024 ± 0.0083 | 0.0022 ± 0.0087 | 0.522 | NS | NS | NS |
| Neutrophils | 0.3144 ± 0.122 | 0.3045 ± 0.0761 | 0.335 ± 0.0722 | 0.227 | NS | NS | NS |

*** Values are reported as mean proportion +- standard error.**

**** Not significant**
