# Supplementary material for: Linking the effects of helminth infection, diet and the gut microbiota with human whole-blood signatures
Source: PLoS Pathog. 2019 Dec 16;15(12):e1008066. doi: 10.1371/journal.ppat.1008066 (PMC6913942; doi:10.1371/journal.ppat.1008066)
Supplement: S6 Table — (DOCX) [file ppat.1008066.s018.docx]

**Table S6. Blood and dietary variables and their correlation with pre-deworming *Trichuris* egg counts**

| Covariate | Pearson correlation | P-value | FDR |
| --- | --- | --- | --- |
| Alkaline_Phosphatase | 0.72 | 1.47E-10 | 0.0% |
| Dietary_Fiber | -0.67 | 1.24E-08 | 0.0% |
| Phosphorus | 0.57 | 3.18E-06 | 0.0% |
| Zn | 0.52 | 3.05E-05 | 0.1% |
| HDL | -0.51 | 3.67E-05 | 0.1% |
| Alpha_Tocopherol | -0.49 | 1.11E-04 | 0.3% |
| Phosphorus2 | 0.48 | 1.19E-04 | 0.4% |
| Platelet | 0.47 | 1.70E-04 | 0.5% |
| T_Bilirubin | -0.47 | 1.70E-04 | 0.5% |
| Saturated_Fat | -0.47 | 1.84E-04 | 0.5% |
| Folate | -0.47 | 1.86E-04 | 0.5% |
| Sugar_Total | -0.46 | 2.68E-04 | 0.7% |
| MFA_Oleic | -0.46 | 2.72E-04 | 0.7% |
| Calsium2 | -0.46 | 3.06E-04 | 0.7% |
| Meat_Lean | 0.45 | 4.18E-04 | 0.9% |
| Fat_Total | -0.45 | 4.23E-04 | 0.9% |
| Bread_Starch | 0.42 | 9.61E-04 | 1.9% |
| Vitamin_K | -0.42 | 1.16E-03 | 2.2% |
| Sodium2 | -0.41 | 1.40E-03 | 2.5% |
| Fruit | -0.40 | 2.02E-03 | 3.4% |
| PFA_DHA | -0.39 | 2.29E-03 | 3.7% |
| MCV | -0.39 | 2.60E-03 | 3.9% |
| Globulin | 0.39 | 2.79E-03 | 3.9% |
| Niacin | 0.38 | 3.12E-03 | 4.1% |
| Serum_Iron | -0.38 | 3.72E-03 | 4.5% |
| A_G_Ratio | -0.37 | 4.36E-03 | 4.8% |
| Eosinophils | 0.35 | 6.37E-03 | 6.4% |
| Vitamin_D | -0.35 | 6.64E-03 | 6.4% |
| Creatinine | -0.34 | 8.19E-03 | 6.5% |
| Selenium | -0.33 | 1.12E-02 | 7.8% |
| Manganese | -0.29 | 2.88E-02 | 17.3% |
| Chromium | -0.26 | 5.06E-02 | 25.3% |
| ESR | -0.23 | 8.40E-02 | 33.6% |
| PCV | -0.18 | 1.76E-01 | 52.7% |
| Hb | -0.12 | 3.71E-01 | 74.2% |
| MCHC | -0.01 | 9.51E-01 | 95.1% |
